# Differential and organ-specific functions of organic solute transporter α and β in experimental cholestasis

**DOI:** 10.1016/j.jhepr.2022.100463

**Published:** 2022-03-05

**Authors:** Sandra M.W. van de Wiel, Begoña Porteiro, Saskia C. Belt, Esther W.M. Vogels, Isabelle Bolt, Jacqueline L.M. Vermeulen, D. Rudi de Waart, Joanne Verheij, Vanesa Muncan, Ronald P.J. Oude Elferink, Stan F.J. van de Graaf

**Affiliations:** 1 Tytgat Institute for Liver and Intestinal Research, Amsterdam UMC, University of Amsterdam, the Netherlands; 2 Amsterdam Gastroenterology Endocrinology Metabolism, Amsterdam, the Netherlands; 3 CIMUS, Universidade de Santiago de Compostela-Instituto de Investigación Sanitaria, Santiago de Compostela, 15782, Spain; 4 Department of Pathology, Amsterdam UMC, University of Amsterdam, the Netherlands; 5 Department of Gastroenterology and Hepatology, Amsterdam UMC, University of Amsterdam, the Netherlands

**Keywords:** ASBT, NTCP, BSEP, Cholestasis, SLC51A, diarrhea, FXR, bile acid, AAV8, adeno-associated virus serotype 8, ALP, alkaline phosphatase, ALT, alanine aminotransferase, ASBT, apical sodium-dependent bile acid transporter, AST, aspartate aminotransferase, BDL, bile duct ligation, CDX2, caudal type homeobox 2, DDC, 3.5-diethoxycarbonyl-1.4-dihydrocollidine, FGF, fibroblast growth factor, FXR, farnesoid X receptor, OST, organic solute transporter

## Abstract

**Background & Aims:**

Organic solute transporter (OST) subunits OSTα and OSTβ facilitate bile acid efflux from the enterocyte into the portal circulation. Patients with deficiency of OSTα or OSTβ display considerable variation in the level of bile acid malabsorption, chronic diarrhea, and signs of cholestasis. Herein, we generated and characterized a mouse model of OSTβ deficiency.

**Methods:**

*Ostβ*^-/-^ mice were generated using CRISR/Cas9 and compared to wild-type and *Ostα*^-/-^ mice. OSTβ was re-expressed in livers of *Ostβ*^-/-^ mice using adeno-associated virus serotype 8 vectors. Cholestasis was induced in both models by bile duct ligation (BDL) or 3.5-diethoxycarbonyl-1.4-dihydrocollidine (DDC) feeding.

**Results:**

Similar to *Ostα*^-/-^ mice, *Ostβ*^-/-^ mice exhibited elongated small intestines with blunted villi and increased crypt depth. Increased expression levels of ileal Fgf15, and decreased Asbt expression in *Ostβ*^-/-^ mice indicate the accumulation of bile acids in the enterocyte. In contrast to *Ostα*^-/-^ mice, induction of cholestasis in *Ostβ*^-/-^ mice by BDL or DDC diet led to lower survival rates and severe body weight loss, but an improved liver phenotype. Restoration of hepatic Ostβ expression via adeno-associated virus-mediated overexpression did not rescue the phenotype of *Ostβ*^-/-^ mice.

**Conclusions:**

OSTβ is pivotal for bile acid transport in the ileum and its deficiency leads to an intestinal phenotype similar to *Ostα*^-/-^ mice, but it exerts distinct effects on survival and the liver phenotype, independent of its expression in the liver. Our findings provide insights into the variable clinical presentation of patients with OSTα and OSTβ deficiencies.

**Lay summary:**

Organic solute transporter (OST) subunits OSTα and OSTβ together facilitate the efflux of conjugated bile acids into the portal circulation. Ostα knockout mice have longer and thicker small intestines and are largely protected against experimental cholestatic liver injury. Herein, we generated and characterized Ostβ knockout mice for the first time. Ostα and Ostβ knockout mice shared a similar phenotype under normal conditions. However, in cholestasis, Ostβ knockout mice had a worsened overall phenotype which indicates a separate and specific role of OSTβ, possibly as an interacting partner of other intestinal proteins.

## Highlights

• This manuscript describes the first mouse model of OSTβ deficiency.
• *Ostβ*^-/-^ mice are viable and fertile, but show increased length and weight of the small intestine, blunted villi and deeper crypts.
• Ostβ deficiency leads to an altered microbiome compared to both wild-type and *Ostα*^-/-^ mice.
• Cholestasis led to lower survival and worse body weight loss, but an improved liver phenotype, in *Ostβ*^-/-^ mice compared to *Ostα*^-/-^ mice.

## Graphical abstract

**Figure:**
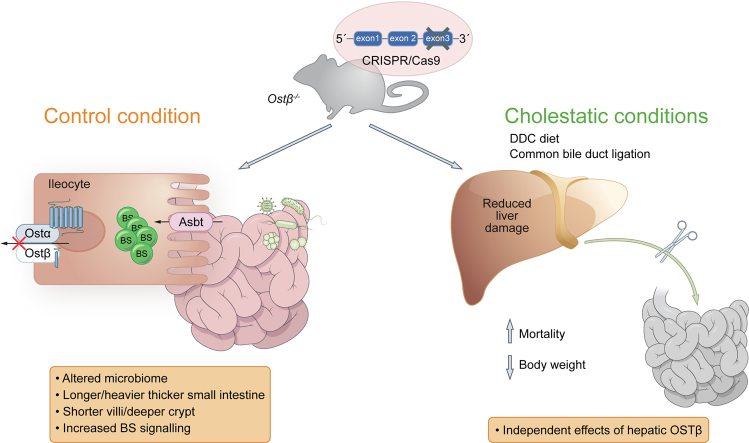

## Introduction

Bile acids facilitate the intestinal digestion and absorption of fats and fat-soluble vitamins. Bile acids are synthesized in hepatocytes from cholesterol via several enzymatic steps that form the primary bile acids cholic acid and chenodeoxycholic acid. The first and rate-limiting step of this cascade is mediated by CYP7A1. Bile acids are subsequently conjugated with amino acids glycine and taurine to form glycocholic acid, taurocholic acid, glycochenodeoxycholic acid and taurochenodeoxycholic acid.^1^ A portion of primary bile acids are converted into the secondary bile acids deoxycholic acid, lithocholic acid and ursodeoxycholic acid by gut bacteria in the intestine.^1^ Compared to humans, mice have a more hydrophilic bile acid composition as they can also synthesize (α-, β- or Ω-) muricholic acid from chenodeoxycholic acid.

Tight regulation of bile acid homeostasis prevents intracellular accumulation of toxic bile acids, which can disrupt membranes, and lead to generation of reactive oxygen species and initiation of apoptosis.^2^ The nuclear farnesoid X receptor (FXR) plays a central role in regulating several genes involved in the enterohepatic circulation of bile acids. Intestinal FXR increases gene expression of fibroblast growth factor (FGF)19, the human homolog of mouse FGF15, upon binding by bile acids.^3^ FGF15/19 is released by the enterocyte into the portal circulation and binds to the FGF receptor 4 (FGFR4)-β-Klotho complex on hepatocytes, which triggers several pathways including the suppression of the rate-limiting enzyme in bile acid synthesis, CYP7A.^3^ In addition, activation of FXR also protects against bile acid overload in both enterocytes and hepatocytes. This is achieved by inhibiting bile acid influx via downregulation of the apical sodium-dependent bile acid transporter (ASBT) and the hepatic uptake transporter sodium taurocholate cotransporting polypeptide and stimulating export of bile acids by upregulation of efflux transporters, such as the bile salt export pump and the organic solute transporter α-β (OSTα-OSTβ).^4^

OSTα-OSTβ transports conjugated bile acids across the basolateral membrane of enterocytes into the portal circulation.[5], [6], [7] This transporter is a heterodimer that consists of 2 distinct subunits; α and β,^8^ encoded by 2 different genes, *SLC51A* and *SLC51B*, located on separate chromosomes. The α-subunit consists of 340 amino acids with 7 transmembrane domains, while the beta-subunit only has 128 amino acids and includes 1 transmembrane domain.^6^ Heterodimerization of the 2 subunits leads to increased stability of the proteins and is necessary for plasma membrane trafficking and transport activity.^9^

OSTα-OSTβ functions in cellular efflux of both conjugated bile acids and steroid hormones, independently of the sodium gradient.^7^ Moreover, *in vitro* studies show that OSTα-OSTβ is able to mediate both cellular efflux and influx, dependent on the concentration gradient of the substrate and extracellular pH.^7^ Highest expression levels of OSTα-OSTβ are detected in the distal part of the ileum. However, OSTα-OSTβ also shows expression in other tissues involved in bile acid homeostasis, such as the kidney and liver, and tissues involved in steroid hormone homeostasis.^7^ Of note, OSTα and OSTβ are expressed with highly varying protein ratios and their transcriptional regulation is poorly correlated.^7^ The relevance of this is not yet known.

To elucidate the physiological role and pathophysiological implications of OSTα deficiency, *Ostα*^*-/-*^ mice have previously been generated.[10], [11], [12], [13], [14] Knockout of the *Ostα* gene leads to complete loss of the OSTα protein, strongly reduced OSTβ,^10^^,^^11^^,^^13^ and results in impaired intestinal bile acid absorption and bile acid accumulation in enterocytes.^11^ Compared to control mice, *Ostα*^*-/-*^ mice display an ameliorated liver phenotype upon bile duct ligation (BDL), and this has been attributed to increased urinary bile acid excretion.^14^ Bile acid accumulation and associated histological changes in the intestine are prevented in *Ostα*^*-/-*^ mice that also lack *Asbt* while *Fxr* depletion did not resolve the phenotype of *Ostα*^*-/-*^ mice. While mutations in the *Asbt* gene are known to cause bile acid malabsorption in humans,^15^ genetic defects in *Asbt* do not account for all hereditary cases of bile acid malabsorption.^16^ In 2019, 2 brothers were identified with a frameshift mutation in the *OSTβ*/*SLC51B* gene causing impaired bile acid transport activity.^17^ These patients had diarrhea, fat-soluble vitamin deficiencies and features of cholestasis, including moderately increased levels of the liver enzymes alanine aminotransferase (ALT), aspartate aminotransferase (AST) and gamma-glutamyltransferase (GGT).^17^ Due to the limited availability of biospecimens from these 2 patients, little is known about the consequence of OSTβ deficiency in humans. Recently, the first OSTα-deficient patient was identified; this patient had diarrhea and cholestasis,^18^ which is not observed in *Ostα*^*-/-*^ mice.^11^^,^^14^ The OSTα-OSTβ complex has an overall topology similar to the heteromeric structure of G-protein coupled receptors associated to receptor activity-modifying proteins^9^^,^^19^ where OSTα adopts a 7-pass transmembrane structure, and OSTβ is a transmembrane protein that crosses the membrane once. OSTβ expression is necessary for glycosylation and trafficking of OSTα to the plasma membrane as well as for functional bile acid transport,^5^^,^^9^^,^^20^ but whether its function is restricted to this chaperone function is unknown. Therefore, an OSTβ knockout mouse model was generated to study the role of OSTβ and to analyze whether deficiency of *Ostβ* in mice affects cholestatic liver injury.

## Materials and methods

For further details regarding the materials and methods used, please refer to the CTAT table and supplementary information.

### Animals

*Ostβ*^*-/-*^ mice were generated in C57BL/6J mice by precise targeted deletion via CRISPR/Cas9, which resulted in a large deletion in exon 3 of the *Ostβ (Slc51b)* gene. To this end, 2 single-guide RNA (sgRNA) target sequences in the *Ostβ* gene were selected and inserted in a pDR274 gRNA cas9-guide plasmid. The sgRNA were synthesized *in vitro*, purified and microinjected together with Cas9 mRNA into 1-cell stage wild-type embryos. These mice were backcrossed once to wild-type mice and resulting *Ostβ*^*+/-*^ animals were crossed to create *Ostβ*^*-/-*^ and wild-type littermates for analysis. Sequencing was performed to confirm the exact genotypes of the mutated *Ostβ* gene and to analyze whether mutations occurred in potential off-target genes, which was not the case. *Ostα*^*-/-*^ mice were generated by Rao *et al.*^13^ and purchased from the Jackson Laboratory. Male and female *Ostα*^*-/-*^*, Ostβ*^*-/-*^ and control wild-type C57BL/6J mice (Janvier Labs) were housed under a 12 h light/dark cycle and bred in the Animal Research Institute Amsterdam. Mice were fed with normal chow diet and given *ad libitum* access to water. The study design, animal care and handling were approved by the Institutional Animal Care and Use Committee of the University of Amsterdam (Amsterdam, The Netherlands).

### Cholestatic mice models

Wild-type and *Ostβ*^*-/-*^ female and male adult mice (littermates) 8-12 weeks of age were subjected to a common BDL as previously described.^21^ All surviving mice (both males and females) were sacrificed at day 5 because of animal welfare regulations (body weight loss >15%). A second cohort of male mice, including wild-type, *Ostα*^*-/-*^ and *Ostβ*^*-/-*^ adult (age 20-30 weeks) mice, were sacrificed 2 days after BDL. In a third cohort of mice, cholestasis was induced by supplementing the chow diet (D12450B1, Open Source Diets, USA) with 0.1% 3,5-diethoxycarbonyl-1,4-dihydrocollidine (DDC, Sigma) during 8 days.^22^ In indicated experiments, DDC diet was initiated 2 weeks after administration via the tail vein of 2x10^12^ adeno-associated virus serotype 8 (AAV8) particles/kg encoding codon optimized mouse OSTβ (Vectorbuilder). All mice were sacrificed under anesthesia and blood, bile and tissues were collected as described in the supplementary information.

### Statistical analysis

Data are provided as mean ±SD with individual points shown in dots. Differences between groups were analyzed using a one-way ANOVA test, and Dunnett’s test to compare with the wild-type littermates or Sidak’s multiple comparisons test. Differences in survival were assessed using a log-rank test. Statistical significance was considered at *p* <0.05(∗). Graphs were generated using GraphPad Prism software (version 8.0.2; GraphPad Software Inc.). Differences in microbiota α diversity were tested using ANOVA. Permanova was used to test compositional differences in terms of Bray-Curtis dissimilarity and Weighted Unifrac distances. Differential abundance of taxa was tested using DESeq2.^23^

## Results

### Generation of OSTβ knockout mice

**Fig. 1:**
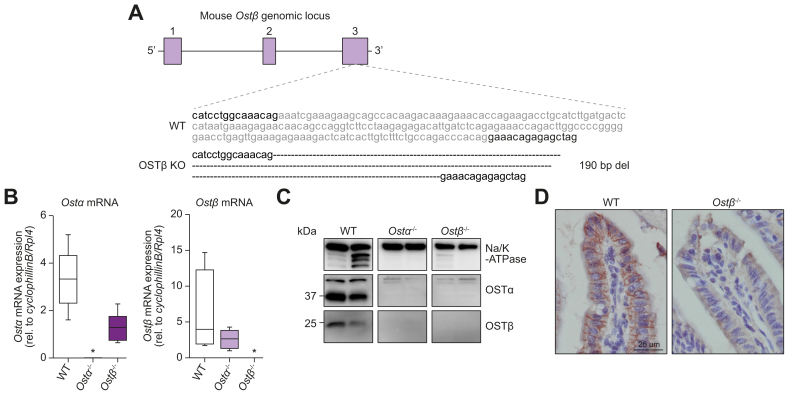
Generation of *Ostβ*^*-/-*^ mice. (A) Schematic representation of the wild-type OSTβ and knockout *OSTβ* gene resulting from CRISPR/Cas9-mediated deletion of exon 3. (B) *OSTα* and *OSTβ* mRNA expression in ileums of 4- and 8-week-old male wild-type, *Ostα*^-/-^ and *Ostβ*^*-/-*^ mice. Data are normalized using the geometric mean of *CyclophillinB* and *Rpl4* (n = 5–7 mice per group). Statistical analysis was done using a one-way ANOVA test and Dunnett’s test to compare with wild-type littermates. ∗Indicates *p* value of <0.05. (C) OSTα and OSTβ protein expression in ileums of 4-week-old female wild-type, *Ostα*^-/-^ and *Ostβ*^*-/-*^ mice. Na/K-ATPase is used as loading control. (D) Immunohistochemistry on ileal sections from wild-type and *Ostβ*^*-/-*^ mice stained with antibody against OSTβ. Original magnification, 400x. Scale bar 25 μm. bp, base pair; del, deletion; KO, knockout; *Ostα*, organic solute transporter alpha; *Ostβ*, organic solute transporter beta; WT, wild-type.

To study the role of OSTβ in mice, targeted deletion was performed using CRISPR-Cas9, resulting in a 190 base pair deletion in exon 3 of the *Ostβ* gene (Fig. 1A). *Ostα* and *Ostβ* mRNA were not expressed in *Ostα*^*-/-*^ and *Ostβ*^*-/-*^ mice, respectively (Fig. 1B). Western blotting confirmed the complete absence of OSTα and OSTβ protein in *Ostα*^-/-^ and *Ostβ*^*-/-*^ mice, respectively (Fig. 1C). In line with previous *Ostα*^-/-^ studies, we found that *Ostα*^-/-^ mice lack the OSTα protein and have strongly reduced OSTβ protein expression,^10^^,^^11^^,^^13^ while *Ostβ*^*-/-*^ mice lack both the OSTβ protein as well as the OSTα protein. Consistent with the western blot, immunohistochemistry showed protein expression of OSTβ on the basolateral membrane of ileal enterocytes in wild-type mice, while this signal was absent in *Ostβ*^*-/-*^ mice (Fig. 1D).

### Phenotype of *Ostα*^-/-^ and *Ostβ*^-/-^ mice

**Fig. 2:**
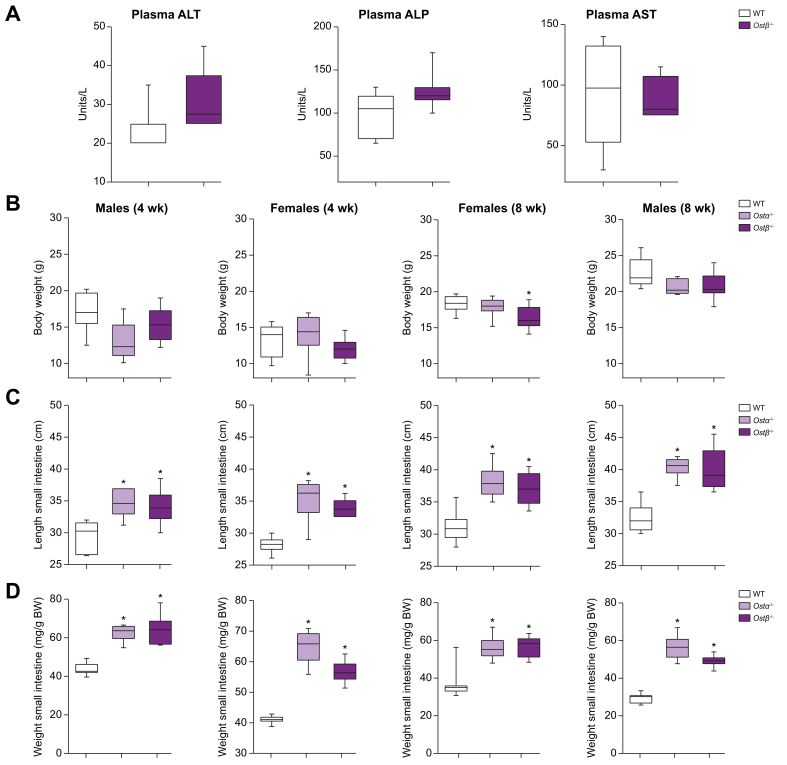
The phenotype of *Ostα*^*-/-*^ and *Ostβ*^*-/-*^ mice compared with wild-type littermates at 4 and 8 weeks of age in females and males. (A) Plasma ALT, ALP and AST levels of 8–12-week-old male mice. (B) Body weight, (C) length of small intestine and (D) weight of small intestine of 4- or 8-week-old male and female mice. Data are expressed as the mean ±SD with individual points shown in dots (n = 7–13 mice per group). Statistical analysis was done using a one-way ANOVA test and Dunnett’s test to compare with wild-type littermates. ∗Indicates *p* values of <0.05. ALP, alkaline phosphatase; ALT, alanine aminotransferase; AST, aspartate aminotransferase; BW, body weight; *Ostα*, organic solute transporter alpha; *Ostβ*, organic solute transporter beta; wk, week.

Both *Ostα*^-/-^ and *Ostβ*^*-/-*^ mice are viable and showed no obvious change in appearance and growth. Crossing heterozygous *Ostβ*^*+/-*^ mice produced a Mendelian distribution of wild-type and knockout genotypes. In contrast to the OSTβ-deficient patients, *Ostβ*^*-/-*^ mice showed no signs of diarrhea. Only a trend towards a modestly increased plasma level of the liver enzymes ALT (*p =* 0.073) and alkaline phosphatase (ALP; *p =* 0.075) was detected and AST levels were unchanged (Fig. 2a). *Ostα*^-/-^ mice showed no significant change in body weight at 4 and 8 weeks after birth in both females and males. Likewise, *Ostβ*^*-/-*^ mice did not demonstrate altered body weight except for 8-week-old females that showed a modest reduction in body weight compared to wild-type littermates (Fig. 2B). The length and weight of the small intestine were significantly and similarly increased in the *Ostα*^-/-^ and *Ostβ*^*-/-*^ mice in both 4- and 8-week-old mice (Fig. 2C,D). The weight per length had a tendency to increase in the *Ostα*^-/-^ and *Ostβ*^*-/-*^ mice that were 4 weeks of age, and was significantly increased in 8-week-old male mice and female *Ostα*^-/-^ mice (Fig. S1A). Liver weight and kidney weight were not changed in the *Ostα*^*-/-*^ and *Ostβ*^*-/-*^ mice (Fig. S1B,C). The length, weight and weight per length of the colon were not altered in *Ostα*^-/-^ and *Ostβ*^*-/-*^ mice (Fig. S1D-F). The small intestine phenotype was preserved in older *Ostβ*^*-/-*^ mice (32-37 weeks) (Fig. S1G).

### Altered ileal histology in *Ostα*^*-/-*^ and *Ostβ*^*-/-*^ mice

**Fig. 3:**
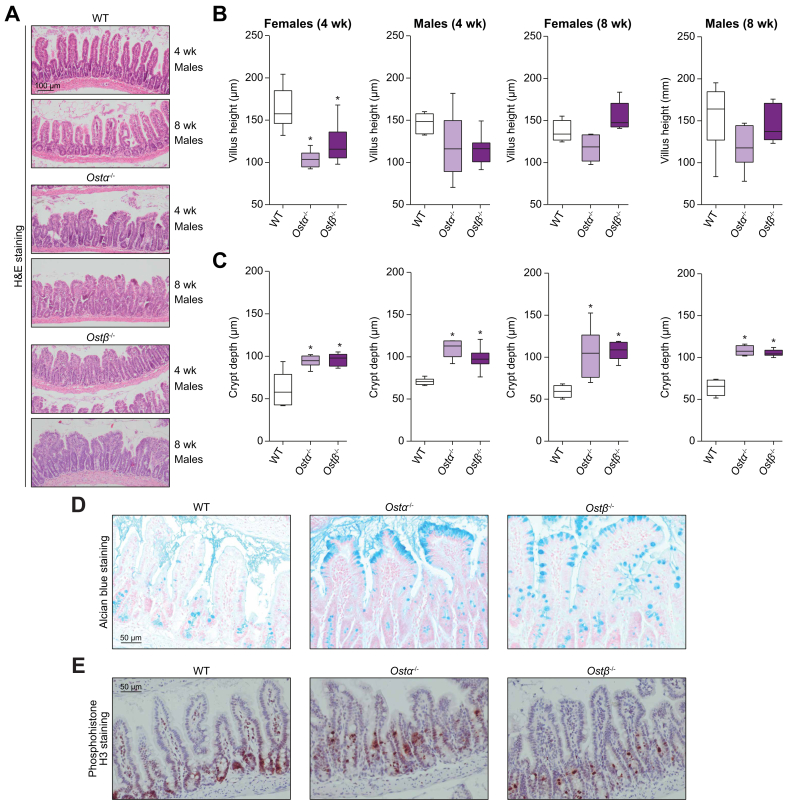
The phenotype of *Ostα*^*-/-*^ and *Ostβ*^*-/-*^ mice compared with wild-type littermates at 4 and 8 weeks of age in females and males. (A) Representative microscopic pictures of H&E-stained transverse sections of the distal ileum (n = 4-8 per group) in male mice. Original magnification, 100x. Scale bar 100 μm. (B-E) Quantitative analysis of ileal sections measured from 5 field views per mouse (n = 4-8). (B) Villus height presented as mean ±SD with individual points showing the mean villus height per mouse (30-90 villi) (C) Crypt depth presented as mean ±SD with individual points showing the mean crypt depth per mouse (30-90 villi). (D) Representative microscopic picture of Alcian Blue staining on paraffin-embedded ileal sections from *Ostα*^*-/-*^*, Ostβ*^*-/-*^ and wild-type 8-week-old female mice (n = 3). Original magnification, 200x. Scale bar 50 μm. (E) Representative pictures of phosphohistone H3 staining on ileal sections from *Ostα*^*-/-*^*, Ostβ*^*-/-*^ and wild-type 8-week-old female mice (n = 3). Original magnification, 100x. Scale bar 100 μm. Statistical analysis was performed using a one-way ANOVA test, and Dunnett’s test to compare with wild-type littermates. ∗Indicates *p* values of <0.05. H&E, hematoxylin and eosin; *Ostα*, organic solute transporter alpha; *Ostβ*, organic solute transporter beta; wk, week; WT, wild-type.

Analysis of the ileum showed an altered histology in *Ostα*^*-/-*^ and *Ostβ*^*-/-*^ male and female mice (Fig. 3A, Fig. S2A). While the ileum of wild-type mice comprises normal-appearing long, thin villi, *Ostα*^*-/-*^ and *Ostβ*^*-/-*^ mice exhibit blunted villi and elongated crypt depth. This altered ileal histology is similar between *Ostα*^*-/-*^ and *Ostβ*^*-/-*^ mice. Quantification of villus height showed a 25% reduction in 4-week-old female and a (not significant) 21% reduction in male *Ostβ*^*-/-*^ mice compared to wild-type littermates (female mice: *Ostβ*^*-/-*^ 121.7 μm ± 24.45 *vs.* wild-type 163.4 μm ± 25.20) (male mice: *Ostβ*^*-/-*^ 115.5 μm ± 17.66 *vs.* wild-type 146.8 μm ± 12.20) (Fig. 3B). Additionally, crypt depth was significantly increased at both 4 weeks of age by 57% and 38% in female and male *Ostβ*^*-/-*^ mice respectively, and 8 weeks of age by 83% and 64% in female and male *Ostβ*^*-/-*^ mice respectively (Fig. 3C). As a result of the increased crypt depth and decreased villus height, the ratio was significantly decreased in *Ostα*^*-/-*^ and *Ostβ*^*-/-*^ mice (Fig. S2B). The top of the ileal villi of *Ostα*^*-/-*^ and *Ostβ*^*-/-*^ mice have increased numbers of mucus-filled vacuoles (Fig. 3D). Furthermore, intestinal proliferation was determined using phosphohistone H3 staining and demonstrated a more widespread distribution along the villi in both *Ostα*^*-/-*^ and *Ostβ*^*-/-*^ mice compared to wild-type mice probably due to the increased crypt depth (Fig. 3E). Other parts of the small intestine, the duodenum and jejunum, were not histologically altered.

### Altered expression of differentiation markers in enterocytes of *Ostα*^*-/-*^ and *Ostβ*^*-/-*^ mice

**Fig. 4:**
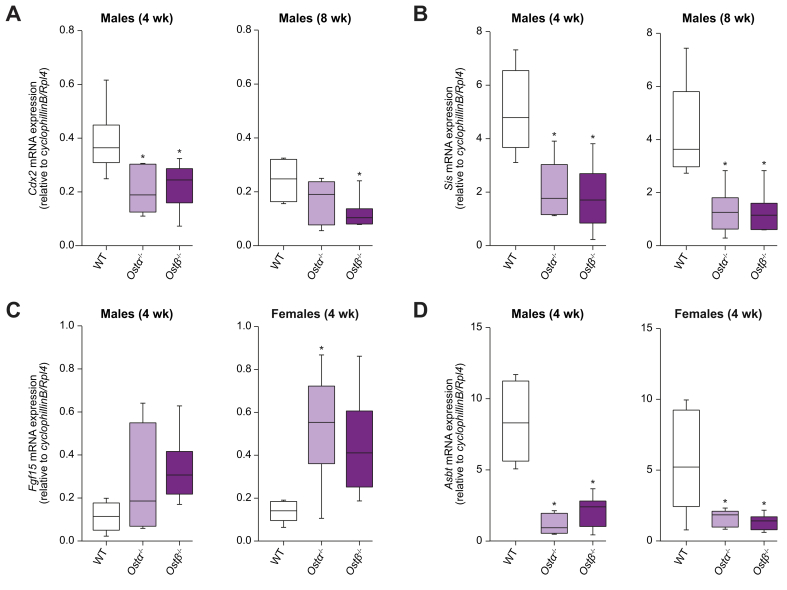
Altered expression of differentiation markers in *Ostα*^*-/-*^ and *Ostβ*^*-/-*^ mice. Ileal mRNA expression of 4- and 8-week-old *Ostα*^*-/-*^, *Ostβ*^*-/-*^ and wild-type males of (A) *Cdx2*, (B) *Sis* (C) *Fgf15* and (D) *Asbt*. Data are normalized using the geometric mean of *CyclophillinB* and *Rpl4*. Data are shown as the mean ±SD with individual points shown in dots (n = 4-7). Statistical analysis was performed using a one-way ANOVA test, and Dunnett’s test to compare with wild-type littermates. ∗Indicates *p* values of <0.05. *Asbt*, apical sodium-dependent bile acid transporter; *Cdx2*, caudal type homeobox 2; *Fgf15*, fibroblast growth factor 15; *Ostα*, organic solute transporter alpha; *Ostβ*, organic solute transporter beta; *Sis*, sucrose-isomaltase; wk, week; WT, wild-type.

Caudal type homeobox 2 (CDX2) induces transcription of several genes implicated in intestinal differentiation and epithelial cell maturation.[24], [25], [26] *Ostβ*^*-/-*^ mice showed a significant decrease in expression of *Cdx2* in the ileum in males at 4 and 8 weeks of age (41% and 50% reduction, respectively), and a similar decreased trend is seen in 4-week-old *Ostβ*^*-/-*^ females (−31%; *p =* 0.15) (Fig. 4A, Fig. S3A). *Ostα*^*-/-*^ and *Ostβ*^*-/-*^ mice showed no change in mRNA expression of *Muc2*, a marker for goblet cells, and *Lysozyme*, a marker for Paneth cells (Fig. S3E,F). However, 4-week-old female and male *Ostβ*^*-/-*^ mice had a significantly decreased expression of sucrase-isomaltase (*Sis*; 65% and 63% reduction, respectively) (Fig. 4B, Fig. S3B) which was confirmed by determining SIS protein levels by immunostaining (Fig. S3C). The change in *Sis* expression was still observed in 8-week-old male mice (∼70% reduction; *p =* 0.0019 and 0.0013), but not in female mice (−25%; *p =* 0.62 and -34%; *p =* 0.74 for *Ostα*^*-/-*^ and *Ostβ*^*-/-*^ mice, respectively). Similarly, mRNA expression of *Arginase2* had a tendency to decrease in *Ostβ*^*-/-*^ mice at both 4 and 8 weeks of age (Fig. S3D). Neonatal markers *Ass1* and *Lct* were not changed at mRNA levels (Fig. S3G,H). Similar findings were obtained in *Ostα*^*-/-*^ mice. Together this indicates that deficiency of OSTβ but also OSTα mainly affects epithelial cells in villi leading to incomplete differentiation.

### Bile salt-related gene expression changes in ileal enterocytes of *Ostα*^-/-^ and *Ostβ*^-/-^ mice

**Fig. 5:**
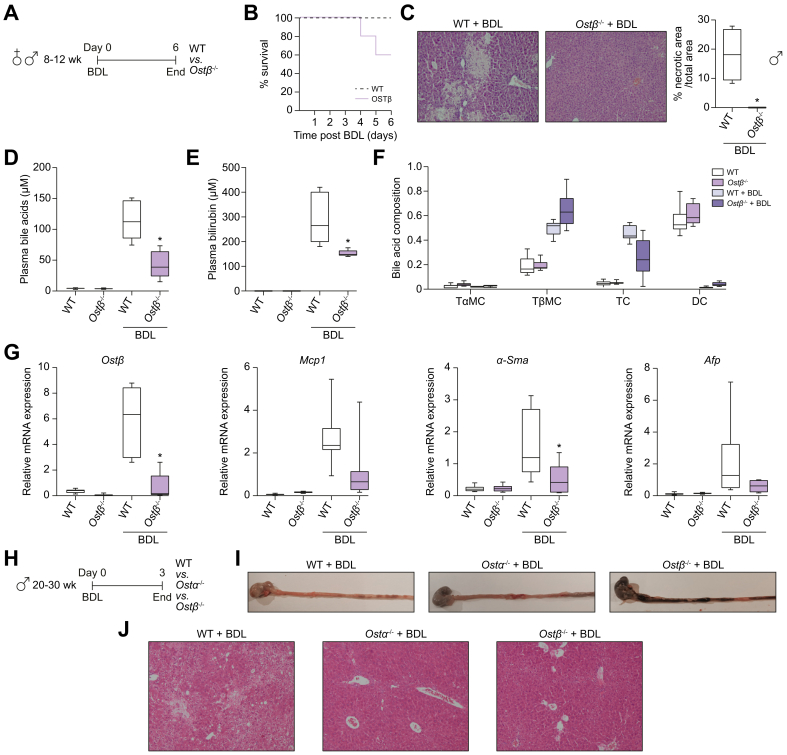
Challenging adult wild-type, *Ostα*^*-/-*^ and *Ostβ*^*-/-*^ mice by inducing cholestasis using common BDL. (A) Schematic representation of the experimental design. (B) Survival rate of adult female and male *Ostβ*^*-/-*^ mice and wild-type littermates after subjecting them to common BDL (n = 7–10). (C) Representative liver microscopic pictures of H&E-stained transverse sections (n = 3–4) per group in male mice after 5 days BDL. Areas of necrosis are indicated and quantified (right panel). Plasma levels of (D) bile acids and (E) bilirubin. (F) Plasma bile acid composition. (G) Hepatic mRNA levels of *Ostβ, Mcp1, α-Sma* and *Afp* in adult female and male *Ostβ*^*-/-*^ mice and wild-type littermates 5 days after BDL. (H) Schematic representation of the experimental design. (I) Representative pictures of the stomach and the first 10 cm of the small intestine in adult male wild-type (n = 1), *Ostα*^*-/-*^ (n = 3) and *Ostβ*^*-/-*^ (n = 3) mice sacrificed 2 days after BDL. (J) Representative liver microscopic pictures of H&E-stained transverse sections (n = 1–3) per group in male mice after 2 days BDL. Original magnification, 100x. Scale bar 100 μm. Statistical analysis was performed using a one-way ANOVA test, and Dunnett’s test to compare with the wild-type littermates. ∗*p* values of <0.05 were considered statistically significant. *Afp*, alpha-fetoprotein; *α-Sma*, alpha smooth muscle actin; BDL, bile duct ligation; DC, deoxycholic acid; *Mcp1*, monocyte chemoattractant protein-1; Ostα, organic solute transporter alpha; *Ostβ*, organic solute transporter beta; TαMC, tauro-alpha-muricholic acid; TβMC, tauro-beta-muricholic acid; TC, taurocholate; WT, wild-type.

Gene expression levels of *Fabp6, Fgf15*, *Mrp3* and *Asbt* were measured to assess possible adaptations related to bile acid transport. *Fabp6* (encoding IBABP) and *Mrp3* mRNA levels were not increased in *Ostα*^*-/-*^ and *Ostβ*^*-/-*^ mice (Fig. S4A,B,E,F). In contrast, *Fgf15* levels increased 3.8-fold and 2.9-fold in *Ostα*^*-/-*^ females and *Ostβ*^*-/-*^ males at 4 weeks of age. Furthermore, *Fgf15* levels tended to increase 3.2-fold (*p =* 0.056) in 4-week-old female *Ostβ*^*-/-*^ mice and 2.4-fold (*p =* 0.26) in 4-week-old *Ostα*^*-/-*^ males (Fig. 4c). Both *Ostα*^*-/-*^ and *Ostβ*^*-/-*^ mice that were 8 weeks of age did not show increased *Fgf15* levels (Fig. 4A, Fig. 4D). Furthermore, *Ostα*^*-/-*^ and *Ostβ*^*-/-*^ mice show decreased expression of the apical bile acid uptake transporter *Asbt* at both ages, which could serve as a protective mechanism against bile acid overload (Fig. 4D, Fig. S4C). Organoids were cultured from ileal stem cells of the *Ostα*^-/-^, *Ostβ*^*-/-*^ and wild-type mice to investigate whether the altered ileal morphology and gene expression is due to cell-intrinsic factors (Fig. S5A,B). Both *Ostα*^*-/-*^, *Ostβ*^*-/-*^ and wild-type organoids grew in the same manner regarding their size and number of buds (Fig. S5A). Furthermore, expression levels of *Fgf15* and *Ibabp* are similar in *Ostα*^*-/-*^, *Ostβ*^*-/-*^ organoids and wild-type organoids (Fig. 5D,E).

### Bile acid concentration and composition in circulation and excretory systems

Next, we investigated the effect of *Ostβ* deficiency on concentrations and composition in the circulation and excretory systems. *Ostα*^*-/-*^ *and Ostβ*^*-/-*^ mice showed unaltered bile acid concentration and composition in bile or plasma. Furthermore, no increased bile acid excretion in urine or feces was observed and the bile acid hydrophobicity index of bile was unchanged (Fig. S6).

### Decreased β diversity in *Ostβ*^-/-^ microbiome

We evaluated bacterial α and β diversity in *Ostβ*^*-/-*^, *Ostα*^*-/-*^ mice and their wild-type littermates. We found no significant differences in terms of α diversity (a metric of microbial richness) analyzed in 3 different ways (Fig. S7A). In contrast, β diversity showed significant differences in bacterial composition between groups as shown in the principal coordinates analysis plots ((PERMANOVA *p =* 0.001, R2 = 0.19; Fig. S7B). Weighted Unifrac analysis, which takes the relatedness of the microbes into account, did not show significant differences, indicating the microbiota are more similar at higher taxonomic ranks. Comparing abundances of taxa in *Ostβ*^*-/-*^ mice with both wild-type mice and *Ostα*^*-/-*^ mice shows a decrease of *Lactobacillus*, various *Lachnospiraceae* and *Candidatus_Saccharimonas* and increase of *Bifidobacteria* and *Faecalibaculum* (Fig. S7C).

### *Ostβ*^*-/-*^ mice show lower survival rates while displaying hepatoprotective effects during BDL-induced liver injury

While OSTβ- and OSTα-deficient patients show features of cholestatic liver injury,^17^^,^^18^ OSTα*-*deficient mice display attenuated liver disease upon induction of cholestasis by ligation of the common bile duct.^14^ Therefore, we wondered whether challenging *Ostβ*^*-/-*^ mice by inducing cholestasis would affect liver injury. To this end, we performed experiments with 2 distinct cholestasis models; common BDL and a 0.1% DDC-containing diet. Both models revealed an unexpected phenotype specifically in *Ostβ*^*-/-*^ mice. First, 18 *Ostβ*^*-/-*^ mice (8 male) and 20 wild-type littermates (10 male; 8-10 weeks of age) were subjected to common BDL (Fig. 5A). While all wild-type mice survived, 40% of the *Ostβ*^*-/-*^ mice died within 5 days (Fig. 5B; *p =* 0.02 log-rank test). No difference in body weight loss was observed in surviving *Ostβ*^*-/-*^ mice compared to wild-type littermates (Fig. S8A). Remarkably, livers of surviving *Ostβ*^*-/-*^ mice were completely devoid of necrotic areas, which covered 15-20% of the area in wild-type mice (Fig. 5C and Fig. S8E). A clear reduction was observed in bile acid levels (62%), plasma bilirubin (42%) and in cholesterol (38%) levels in the surviving *Ostβ*^*-/-*^ mice, while plasma ALT, ALP and AST levels were unchanged (Fig. 5D,E and Fig. S9A,B). Taurobetamuricholic acid levels are increased in *Ostβ*^*-/-*^ mice compared to wild-type littermates (Fig. 5F). In general, expression levels of markers of hepatic inflammation (*Mcp1*; *p* = 0.0163), fibrosis (*Timp* [*p* = 0.093], *α-Sma* [*p* = 0.19], *Col1a1* [*p* = 0.17]) and proliferation (*Afp* [*p* = 0.14]), but not *Cyp7a1* and *IL6* tended towards being reduced in surviving *Ostβ*^*-/-*^ mice compared to wild-type mice after BDL (Fig. 5G, Fig. S8D). The high mortality upon BDL in *Ostβ*^*-/-*^ mice was confirmed in a second experiment with a group of 3 *Ostβ*^*-/-*^ mice (male, 20-30 weeks old). In this experiment we also included wild-type littermates and *Ostα*^*-/-*^ mice (Fig. 5H). On day 3, animals were sacrificed due to severe symptoms of distress, including hunched posture and lethargy, specifically presented by the *Ostβ*^-/-^ mice. Furthermore, the contents of the stomach and the intestines were dark colored and were located throughout the small intestine while the small intestine of both *Ostα*^*-/-*^ mice and wild-type mice showed a normal color and contained less alimentary matter (Fig. 5I and Fig. S8F). Remarkably, the cages of *Ostβ*^*-/-*^ mice contained considerably less feces compared to the cages of *Ostα*^*-/-*^ mice (data not shown). In line with results of the first BDL experiment, examination of the liver suggested a protective effect in both *Ostα*^*-/-*^ and *Ostβ*^*-/-*^ mice with respect to liver damage due to BDL, with obvious pre-necrotic areas in the wild-type animals (Fig. 5J).

### *Ostβ*^*-/-*^ mice show lower body weight gain while displaying hepatoprotective effects when challenged with a DDC diet

**Fig. 6:**
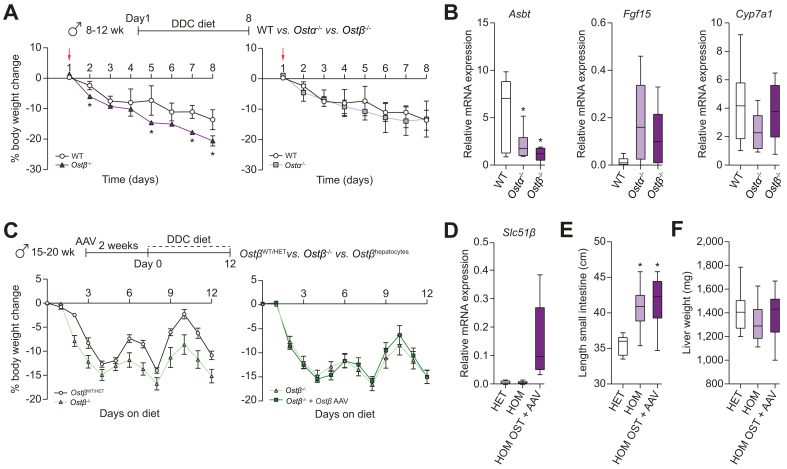
Challenging adult wild-type, *Ostα*^*-/-*^ and *Ostβ*^*-/-*^ mice by inducing cholestasis using a DDC diet. (A) Body weight change in *Ostβ*^*-/-*^ and WT (right panel) and body weight change in *Ostα*^*-/-*^ and WT littermates (left panel) after 8 days with DDC diet (n = 6-9 per group). (B) Ileal mRNA expression of *Asbt*, *Fgf15* and hepatic mRNA expression of *Cyp7a1* (n = 6-9 per group). Data are normalized using the geometric mean of *CyclophillinB*-*Hprt* (ileum) and *Tbp-Hprt* (liver). Adult *Ostβ*^*-/-*^ mice were injected via the tail vein with an AAV8 vector encoding mouse OSTβ and then cholestasis was induced using a DDC diet. (C) Body weight change in *Ostβ*^*-/-*^^WT/HET^ mice and *Ostβ*^*-/-*^ (left panel) and *Ostβ*^*-/-*^ and *Ostβ*^*-/-*^ AAV8 (right panel) after 12 days of a DDC diet (n = 8-10 mice per group). (D) Hepatic mRNA expression of *Slc51β*; data are normalized using the geometric mean of *CyclophillinB-Hprt*. (E) Small intestine length and (F) liver weight of adult *Ostβ*^WT/HET^ mice, *Ostβ*^-/-^ mice and *Ostβ*^-/-^ AAV8 were measured (n = 8–10 mice per group). Statistical analysis was performed using a one-way ANOVA test, and Dunnett’s test to compare with the wild-type littermates. ∗*p* values of <0.05 were considered statistically significant. AAV8, adeno-associated virus serotype 8. AAV, adeno-associated virus; *Asbt*, apical sodium-dependent bile acid transporter; *Cyp7a1*, Cytochrome P450 Family 7 Subfamily A Member 1; DDC diet, 3.5-diethoxycarbonyl-1.4-dihydrocollidine diet; *Fgf15*, fibroblast growth factor 15; HET, heterozygous; HOM, homozygous; *Ostα*, organic solute transporter alpha; *Ostβ*, organic solute transporter beta; *Slc51β*, solute carrier family 51, beta subunit; wk, week.

After 8 days on a DDC diet, *Ostβ*^*-/-*^ mice showed marked body weight loss compared to wild-type and *Ostα*^*-/-*^ mice (Fig. 6A). In contrast, *Mcp-1* levels were significantly lower in *Ostβ*^*-/-*^ mice when compared with wild-type littermates and a significant reduction was found in AST in *Ostα*^*-/-*^ and *Ostβ*^*-/-*^ mice when compared with wild-type mice (Fig. S9A,B) while plasma bilirubin, ALP and ALT levels as well as *α-Sma*, *Col1a1* and *Afp* expression remained unchanged (Fig. S9B). Intestinal *Asbt* expression was decreased and *Fgf15* expression increased in *Ostα*^*-/-*^ and *Ostβ*^*-/-*^ mice also under these cholestatic conditions (Fig. 6B). The discrepancy between overall health status and (selected) markers for liver damage mimics the BDL phenotype and suggests that, in *Ostβ*^*-/-*^ mice, an extrahepatic phenotype is unmasked under cholestatic conditions which is distinct from *Ostα*^*-/-*^ mice. The increased weight loss in *Ostβ*^*-/-*^ mice was confirmed in a second DDC-induced cholestasis experiment where we tested the role of hepatic OSTβ (Fig. 6C). To this end, we included a group that received AAV8 encoding mouse *Ostβ* 2 weeks prior to the onset of the diet. A second difference with the first DDC experiment was that we briefly switched to control chow on day 5-6 and 9-10 to allow recovery of body weight and continued with DDC diet afterwards for another 2.5 days. Body weight loss was more severe in *Ostβ*^*-/-*^ mice than wild-type mice (Fig. 6C). Body weight differences across the entire experiment are calculated as area under the curve (%.day) and were -86.07 ± 5.25 in mice expressing endogenous OSTβ and -129.6 ± 10.9 and -127.0 ± 9.34 in DDC-fed *Ostβ*^*-/-*^ mice (respectively mock injected or treated with OSTβ-AAV8) (Fig. 6C). Hepatic expression of OSTβ was confirmed in the latter group (Fig. 6D). Also, cholestatic *Ostβ*^*-/-*^ mice displayed elongated small intestines (irrespective of restored hepatic OSTβ expression) (Fig. 6E), while no difference in liver weight was present (Fig. 6F). This indicates that the increased body weight loss seen in cholestatic *Ostβ*^*-/-*^ mice likely has an extrahepatic origin.

## Discussion

Here, we generated OSTβ-deficient mice and show that disruption of OSTβ results in profound ileal morphological changes. When unchallenged, no major differences are observed between *Ostα*^*-/-*^ and *Ostβ*^*-/-*^ mice and *Ostβ*^*-/-*^ mice are phenocopying *Ostα*^*-/-*^ mice. Our results are mostly in line with previous *Ostα*^*-/-*^ studies,^10^^,^^11^^,^^13^^,^^14^^,^^20^^,^^27^^,^^28^ suggesting that OSTα and OSTβ function in the same manner in bile acid homeostasis under normal conditions. However, under cholestatic conditions, *Ostβ*^*-/-*^ mice have a worsened phenotype, a significant lower survival rate and lower body weight compared to both wild-type and *Ostα*^*-/-*^ mice. This phenotype is independent of hepatic OSTβ expression status. As the contents of the intestine and stomach of *Ostβ*^*-/-*^ mice were dark colored, while *Ostα*^*-/-*^ mice were indistinguishable from wild-type littermates, an intestinal origin of this phenotype is likely. Furthermore, these data indicate that there might be a difference between the function of OSTα and OSTβ.

The OSTβ-deficiency phenotype under cholestatic conditions does not relate to liver damage since the lower survival rates of *Ostβ*^*-/-*^ mice do not seem to correlate with histology and markers of bile acid-induced liver injury. The *Ostβ* knockout mice even showed some level of protection against liver injury during cholestasis by BDL, similar to *Ostα*^*-/-*^ mice. The discrepancy in content in the colon *vs.* the stomach of cholestatic *Ostβ*^*-/-*^ mice may point to an intestinal motility phenotype. Several papers suggest a link between cholestasis and/or altered bile acid signaling and alterations in intestinal transit via 3 possible mechanisms.[29], [30], [31], [32]

First, the endogenous opioid system has been demonstrated to be activated in cholestatic conditions in mice, leading to decreased intestinal transit.^29^ Therefore, induction of cholestasis may reveal or enhance an intestinal mobility phenotype in *Ostβ*^*-/-*^ mice. Second, activation of TGR5, the GPCR for bile acids, is essential for peristalsis and gastric emptying, possibly via induction of glucagon-like peptide-1 secretion.^30^^,^^31^ *Ostβ*^*-/-*^ mice may have reduced TGR5 activation as the bile acid pool is likely reduced due to chronically elevated *Fgf15* expression. Third, NGM282, an FGF15/19 mimetic has prokinetic activity itself.^32^ Chronic overexpression of FGF15, as seen in *Ostα*^*-/-*^ and *Ostβ*^*-/-*^ mice may lead to desensitization of FGFR/KLB, just as chronic FGF23 overexpression desensitizes this receptor complex.^33^ A rapid reduction in FGF15, as would occur during cholestasis may then lower intestinal motility to pathologically relevant levels. Although such mechanisms could contribute to the intestinal phenotype of *Ostβ*^*-/-*^ mice in cholestatic conditions, it remains unclear why this phenotype is not exposed in *Ostα*^*-/-*^ mice, which are largely indistinguishable with regard to bile acid homeostasis. This suggests that OSTβ might have another function besides forming a bile acid efflux transporter upon heterodimerization with OSTα. Early after the cloning of OSTα-OSTβ it was postulated that OSTβ may function as a chaperone or regulatory subunit for other proteins^6^ as the topology of OSTα-OSTβ is similar to that of G-protein coupled receptors associated to receptor activity-modifying proteins.^34^ This may also explain why the regulation of gene expression of these 2 subunits is so different.^7^ For example, hepatic upregulation of OSTβ expression is much higher than that of OSTα in patients with primary biliary cholangitis^35^ and in obstructive cholestasis.^36^ Finally, the modest but evident differences in microbial composition may lead to or reflect differences in intestinal function. *Ostβ*^*-/-*^ mice were more sensitive to the DDC diet than wild-type or *Ostα*^*-/-*^ mice. *Ostβ*^*-/-*^ mice lost significantly more body weight which may be related to the altered microbiota as this could lower the efficiency of energy harvest.^37^ This would also explain why the effect is independent of hepatic OSTβ expression.

Our *Ostβ*^*-/-*^ model made it possible to compare the consequence of OSTβ deficiency and OSTα deficiency in mice but also to compare this to the few individuals described to date with SLC51A or SLC51B deficiency. In contrast to *Asbt*^*-/-*^ mice which show a similar malabsorptive phenotype as patients with an *Asbt* mutation,^15^^,^^38^ *Ostβ*^*-/-*^ mice do not reflect all characteristics of the 2 OSTβ-deficient patients. The OSTβ-deficient brothers suffer from congenital diarrhea and features of cholestasis,^17^ whereas *Ostα*^*-/-*^ and *Ostβ*^*-/-*^ mice do not. Furthermore, the OSTα-deficient patient who was recently identified showed symptoms similar to the OSTβ-deficient brothers, albeit with more severe signs of cholestasis.^18^ While expression of *Ostα* and *Ostβ* is high in human livers, it is marginal in mouse livers under normal circumstances.^7^ This may explain why OSTα- and OSTβ-deficient patients experience liver histological changes and elevated liver enzymes ALT, AST and GGT, while there is only a trend towards a modest increase in ALT and ALP in the *Ostβ*^*-/-*^ mice. Protective mechanisms are initiated in mice with OSTα or OSTβ deficiency to reduce the bile acid load, which likely explains the ameliorated phenotype in older mice,^10^^,^^11^^,^^13^^,^^14^ although the elongated small intestine remains present in aged *Ostβ*^*-/-*^ mice. In addition, mice have a different gut microbiome composition and enzymatic bile acid (re)hydroxylation repertoire leading to a distinct bile acid composition and conjugation.^39^ The mouse bile acid pool is less hydrophobic and toxic which may dampen liver damage and is also much reduced in OSTα- and OSTβ-deficient mice, lowering the level of diarrhea despite the severely affected ileal morphology.

Gene expression of ileal *Fgf15* was increased, inversely correlated with *Asbt* expression in the ileum and Cyp7a1 in the liver, implying accumulation of bile acids in the enterocyte and dampening of bile acid synthesis. Surprisingly, gene expression of *Fabp6* was not elevated, however, conflicting results on gene expression of *Fabp6* have been observed in *Ostα*^*-/-*^ mice before.[11], [12], [13] Short-term inhibition of OSTα-OSTβ *in vivo* leads to increased FXR activation in enterocytes^40^ and it was previously demonstrated that the increase in *Fgf15* expression in *Ostα*^*-/-*^ mice is due to FXR activation. Recent evidence indicates that the ileal histological changes in *Ostα*^*-/-*^ are secondary to enterocyte injury caused by bile acid accumulation, since disruption of *Asbt* in *Ostα*^*-/-*^ mice restores the intestinal phenotype completely.^11^ Even though expression of *Asbt* is partly downregulated, *Ostα*^*-/-*^ and *Ostβ*^*-/-*^ mice are not able to fully restore the ileal morphology, suggesting that bile acid accumulation in enterocytes is still present. Furthermore, expression of ileal *Mrp3* is not increased, supporting the evidence that MRP3 does not have a major role in conjugated bile acid transport.^41^ Finally, *Ostα*^*-/-*^ and *Ostβ*^*-/-*^ organoids do not show an altered phenotype, suggesting that bile acids cause the altered phenotype in the ileum.

In conclusion, OSTα-OSTβ is an important heterodimeric bile acid transporter. Knockout of either *Ostα* or *Ostβ* results in a severe ileal phenotype that is in line with previous *Ostα* knockout studies. During cholestasis, knockout of either *Ostα* or *Ostβ* seems to ameliorate liver damage. However, unlike in *Ostα*^*-/-*^ mice, these beneficial effects are paralleled by an intestinal motility phenotype in *Ostβ*^*-/-*^ mice, potentially contributing to a significantly lower survival rate and higher body weight loss. This is the first evidence that the role of OSTβ differs from OSTα and suggests that OSTβ might also have an additional, unidentified, intestinal function.

## Financial support

SFJ vd Graaf is supported by the 10.13039/501100003246Netherlands Organisation for Scientific Research (VIDI 91713319; VICI 09150182010007) and the 10.13039/501100000781European Research Council (Starting grant 337479). B. Porteiro BP is recipient of a fellowship from Xunta de Galicia (ED481B 2018/050).

## Authors' contributions

SMWvdW, BP, SCB: study concept and design; acquisition of data; analysis and interpretation of data; drafting of the manuscript; EWMV, IB, JLMV, DRdW, JV: technical support; acquisition of data; analysis and interpretation of data; VM, RPJOE, SFJvdG: study concept and design, study supervision, analysis and interpretation of data; critical revision of the manuscript.

## Data availability statement

All raw data are available upon request to the corresponding author.

## Conflict of interest

The authors declare no conflicts of interest that pertain to this work.

Please refer to the accompanying ICMJE disclosure forms for further details.
