# Supplementary material for: Differential and organ-specific functions of organic solute transporter α and β in experimental cholestasis
Source: JHEP Rep. 2022 Mar 5;4(5):100463. doi: 10.1016/j.jhepr.2022.100463 (PMC9019253; doi:10.1016/j.jhepr.2022.100463)
Supplement: Multimedia component 1 [file mmc1.pdf]

Differential and organ-specific functions of organic solute transporter α and β in experimental cholestasis

Sandra M.W. van de Wiel, Begoña Porteiro, Saskia C. Belt, Esther W.M. Vogels, Isabelle Bolt, Jacqueline L.M. Vermeulen, D. Rudi de Waart, Joanne Verheij Vanesa Muncan, Ronald P.J. Oude Elferinkd, Stan F.J. van de Graaf

Table of contents

Supplementary materials and methods...2

Supplementary figures.. 6

Table S1...17

Supplementary references...19

Supplementary materials and methods

Animals

Male and female *Ostα*$^{-/-}$, *Ostβ*$^{-/-}$ and control wild-type C57BL/6J mice (Janvier Labs) were housed with a 12h light/dark cycle and bred in the Animal Research Institute Amsterdam. Mice were fed with normal chow diet with ad libitum access to water. *Ostα*$^{-/-}$ mice were generated by Rao et al. [1] and purchased from the Jackson Laboratory (B6.129S6-Slc51atm1Pda/J, JAX stock #009082, Jackson Laboratory). *Ostβ*$^{-/-}$ mice were generated in C57BL/6J mice by precise targeted deletion via CRISPR/Cas9, which resulted in a large deletion in exon 3 of the OSTβ gene as described below. *Ostα*$^{-/-}$, *Ostβ*$^{-/-}$ and wild-type littermates were bred in the same facility and sacrificed under anesthesia with ketamine (100mg/kg, Alfasan, 1711347-12) and xylazine (10mg/kg, Sedamun, Dechra, 077368). Age at sacrifice is indicated in the legends. Feces were collected from single- or pair-housed mice over a 24 hour period. Blood was collected by heart puncture and plasma was separated by centrifugation at 2000rpm for 10 minutes. The small intestine was cut into three equal segments. Two centimeter of each part was used for determination of RNA and protein expression. The remaining part was cut open with the luminal side facing upward and then rolled up into a Swiss-roll, fixed in 4% PFA at 4°C o/n and stored in 70% ethanol at 4°C. The study design, animal care and handling were approved by the Institutional Animal Care and Use Committee of the University of Amsterdam (Amsterdam, The Netherlands).

Generation of Ostβ knockout mice

The *Slc51b* gene was analyzed for CRISPR/Cas9 target sites and sgRNA target sequences 5'-TAGGCTGCTTCTTTCGATTTCT-3' and 5'-AAACAGAAATCGAAAGAAGCAG-3' were selected using the online CRISPR design tool on the ZiFiT website (http://zifit.partners.org/ZiFiT/). Potential off-target sites for CRISPR-Cas9 were predicted using the Zhang laboratory website (http://crispr.mit.edu). Target sequences were inserted in a pDR274 gRNA cas9-guide plasmid following BSA I restriction enzyme digestion. Plasmids were linearized using the DraI enzyme and subsequently transcribed using the MEGAshortscript T7 kit (Life Technologies). The sgRNA were purified using the MEGAclear kit (Life Technologies). Cas9 mRNA and sgRNAs were

microinjected in one-cell stage wild-type embryos. Resulting mice were backcrossed once to wild-type mice and *Ostβ*$^{+/-}$ animals crossed to create *Ostβ*$^{-/-}$ and wild-type littermates for analysis. Sequencing was performed to confirm the exact genotypes of the mutated *Ostβ* gene and to analyze whether mutations occurred in potential off-target genes, which was not the case.

Histology

Hematoxylin and eosin staining was performed as described earlier [2]. For Alcian Blue staining, slides were deparaffinized and rehydrated using the standard protocol and sections were stained with Alcian Blue (Sigma, A3157) for 30 minutes, washed in MilliQ and counterstained with Nuclear Fast Red (Merck, 5189) for 5 minutes. The average villus height, crypt depth and villus height to crypt depth ratio of the distal ileum were quantified in five field views at a magnification of 100x in a blinded manner using ImageJ (1.50i) with 30-90 villi/crypts per mouse (n=4-8).

Immunohistochemistry

Sections (4.5µm) from Ostα$^{-/-}$, Ostβ$^{-/-}$ and control wild-type mice were deparaffinized and rehydrated. Staining was performed as previously described [3]. The primary antibodies that were used are rabbit anti-OSTβ (1:100, gift from Paul Dawson [4]), goat anti-sucrase-isomaltase (1:200, A-17, Santa Cruz, sc-27603, L0210) and rabbit anti-phosphohistone H3 (1:200, PA5-17869, ThermoFisher). Subsequently, sections were incubated with poly-HRP goat anti-mouse / rabbit (ImmunoLogic, VWRKC-DPVB110HRP, 010818C) or BrightVision poly-HRP anti-goat (ImmunoLogic, VWRKDPVG110HRP, 150910). Sections were stained using Vector NovaRed (HRP) substrate kit (Vector Laboratories, SK-4800), counterstained with haematoxylin and mounted with Vectamount (Vector Laboratories, ZD0104).

Analysis of gene expression by qPCR

Total RNA was isolated from ileum or liver with TRI Reagent according to the manufacturer's instructions (Sigma-Aldrich, T9424-200). Intestinal organoids were generated from ileum and cultured as previously described [3]. RNA from organoids was isolated from three pooled wells using Isolate II RNA Mini Kit (Bioline, IS510-B064990, B10-52073). cDNA was synthesized as previously described using RevertAid Reverse Transcriptase (Fermentas, EP0442) [3]. Gene expression was

measured and normalized to the geometric mean of the most stable reference genes *CyclophillinB* and *Rpl4*, identified by GeNORM (v3.5). The primer sequences are provided in Supplementary Table 1.

Protein expression

Protein isolation was performed with RIPA buffer (50mM Tris-HCl pH=7.4, 150mM NaCl, 1% NP40-substitute, 0,1% SDS) with protease inhibitors (cOmplete™, Mini, EDTA-free Protease Inhibitor Cocktail) in the Tissuelyser LT (Qiagen) for 15 minutes. Samples were separated as described before [2]. The membrane was incubated with rabbit anti-mouse-OSTα (1:1000 diluted in blocking buffer) and anti-mouse OSTβ (1:1000 diluted in blocking buffer) that were both gifts from Paul Dawson (Atlanta, USA) [1, 5], and was subsequently stained with anti-NA/K-ATPase (1:1000 diluted in blocking buffer) that was a gift from Jan Koenderink (Nijmegen, The Netherlands).

Plasma biochemistry and bile acid species determination

Plasma biomarkers for liver injury (ALT, AST) and cholestatic parameters (ALP) were determined by routine clinical biochemistry testing on a Roche Cobas c502/702 analyzer (Roche Diagnostics). Quantification of bile acids in urine, bile, feces and plasma was performed as previously described [6] and hydrophobicity index calculated as described [7].

Cholestatic mice models

Wild-type and *Ostβ*$^{-/-}$ female and male adult mice (littermates) 8-12 weeks of age were subjected to a common bile duct ligation as previously described [8]. All surviving mice (both males and females) were sacrificed at day 5 because of animal welfare regulations (body weight loss >15%). A second cohort of male mice, including wild-type, *Ostα*$^{-/-}$ and *Ostβ*$^{-/-}$ adult (age 20-30 weeks) mice, was sacrificed two days after bile duct ligation. In a third cohort of mice, cholestasis was induced by supplementing the chow diet (D12450B1, Open Source Diets, USA) with 0.1% 3,5-diethoxycarbonyl-1,4-dihydrocollidine (DDC, Sigma) during 8 days [9]. In indicated experiments, DDC diet was initiated 2 weeks after administration via the tail vein of $2*10^{12}$ AAV8 particles/kg encoding codon optimized mouse OST beta (Vectorbuilder). All mice were sacrificed under anesthesia and blood, bile and tissues were collected as described above.

Fecal DNA extraction and 16S amplicon sequencing

DNA was extracted from fecal material using a repeated bead beating protocol [10]. DNA was purified using Maxwell RSC Whole Blood DNA Kit. 16S rRNA gene amplicons were generated using a single step PCR protocol targeting the V3-V4 region [11]. PCR products were purified using Ampure XP beads and purified products were equimolar pooled. The libraries were sequenced using a MiSeq platform using V3 chemistry with 2 × 251 cycles.

Forward and reverse reads were truncated to 240 and 210 bases respectively and merged using USEARCH [12]. Merged reads that did not pass the Illumina chastity filter, had an expected error rate higher than 2, or were shorter than 380 bases were removed. Amplicon sequence variants (ASVs) were inferred for each sample individually from sequences with a minimum abundance of four reads [12]. Unfiltered reads were then mapped against the joint ASV set to determine per sample ASV abundances. Taxonomy was assigned using the RDP bayes classifier [13] and SILVA [14] 16S ribosomal database V132. Microbial counts were rarefied to 25,000 counts per sample.

Statistical analysis

Data are provided as mean ±SD with individual points shown in dots. Differences between groups were analyzed using a One-way ANOVA test, and Dunnett's test to compare with the wild-type littermates or Sidak's multiple comparisons test. Statistical significance was considered at $P < 0.05$(*). Graphs were generated using GraphPad Prism software (version 8.0.2; GraphPad Software Inc.). Microbiota data were analyzed and visualized in R 3.5.2 using Phyloseq [15], Vegan [16], and Picante [17]. Differences in alpha diversity were tested using ANOVA. Permanova was used to test compositional differences in terms of Bray-Curtis dissimilarity and Weighted Unifrac distances. Differential abundance of taxa was tested using DESeq2 [18].

Supplementary figures

Click on the Supplementary Figures to open the embedded high resolution PDF files.

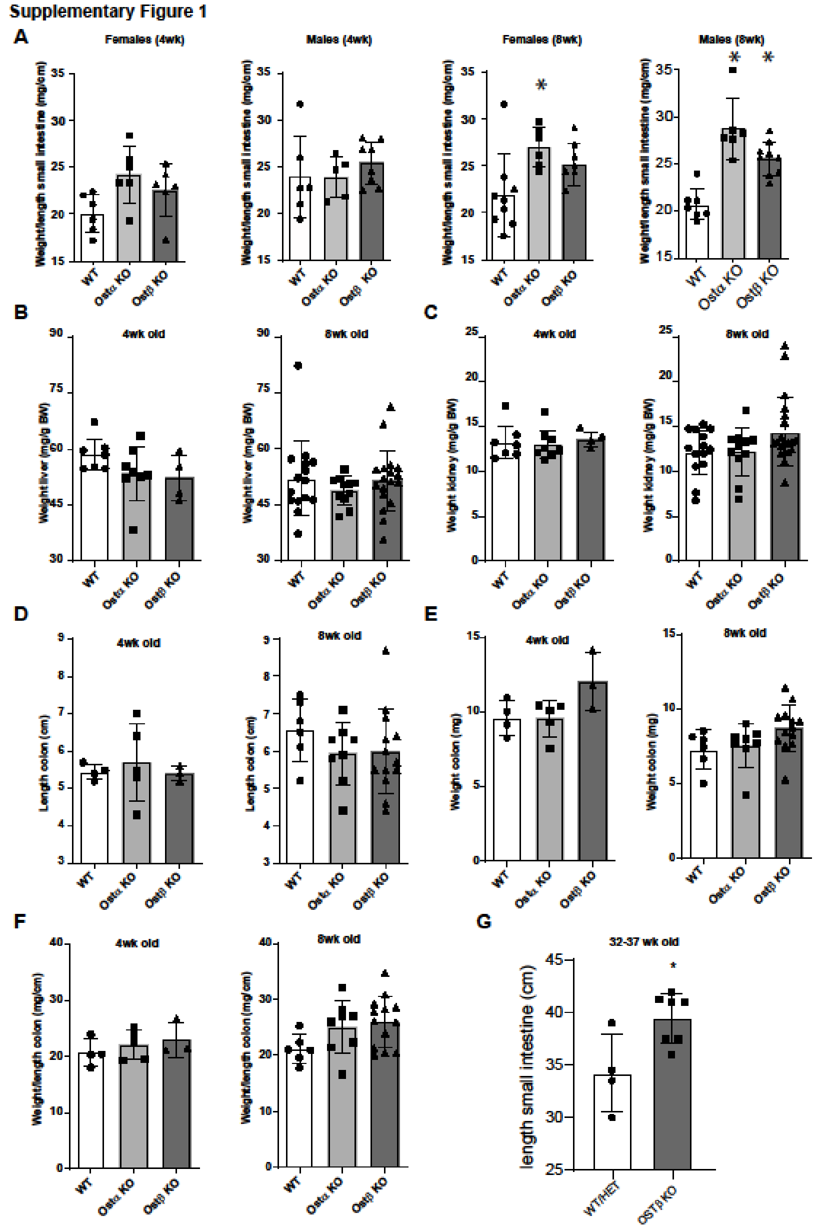

Fig. S1. Phenotype of Ostα$^{-/-}$ and Ostβ$^{-/-}$ mice. (A) Weight per unit length of small intestine of 4 and 8 weeks old male and female mice. (B) Weight of liver, (C) Weight of kidney, (D) Length of colon, (E) Weight of colon and (F) Weight per unit length of colon of 4 and 8 weeks old mice (males and females combined). (G) length of the small intestine in female mice of 32-37 weeks of age showing that some of the intestinal phenotype persists during aging. Data are expressed as the means ±SD with individual points shown in dots (n=3-13 mice per group). Statistical analysis was done using a one-way ANOVA test, and Dunnett's test to compare with the wild-type littermates. *A p-value of <0.05 was considered statistically significant from wild-type mice.

Supplementary Figure 2

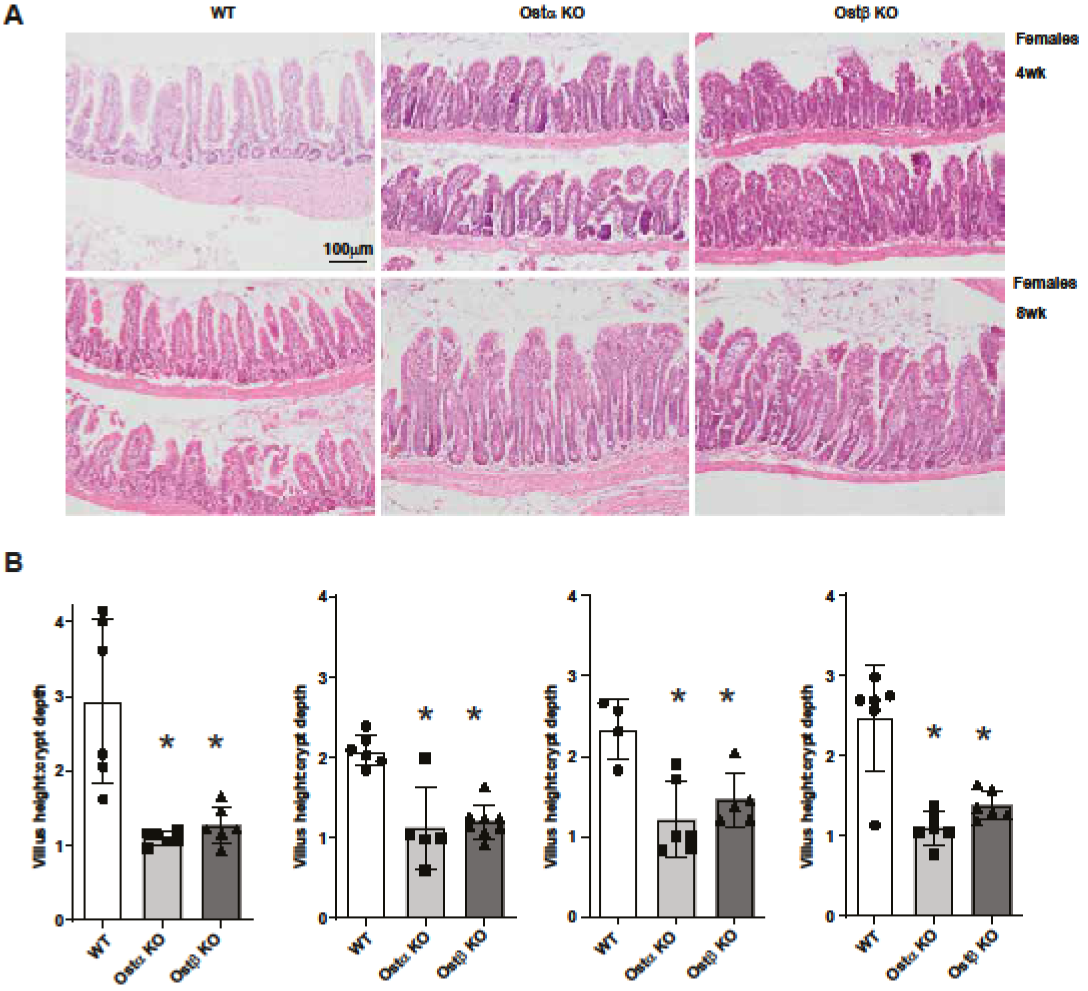

Fig. S2. (A) Representative microscopic pictures of H&E-stained transverse sections of the distal ileum (n=4-8 per group) in female mice. Original magnification, 100x. Scale bar 100µm. (B) Ratio villus height to crypt depth presented as mean ±SD with individual points showing the mean ratio per mouse. Statistical analysis was done using a one-way ANOVA test, and Dunnett's test to compare with the wild-type littermates. *A p-value of <0.05 was considered statistically significant from wild-type mice.

Supplementary Figure 3

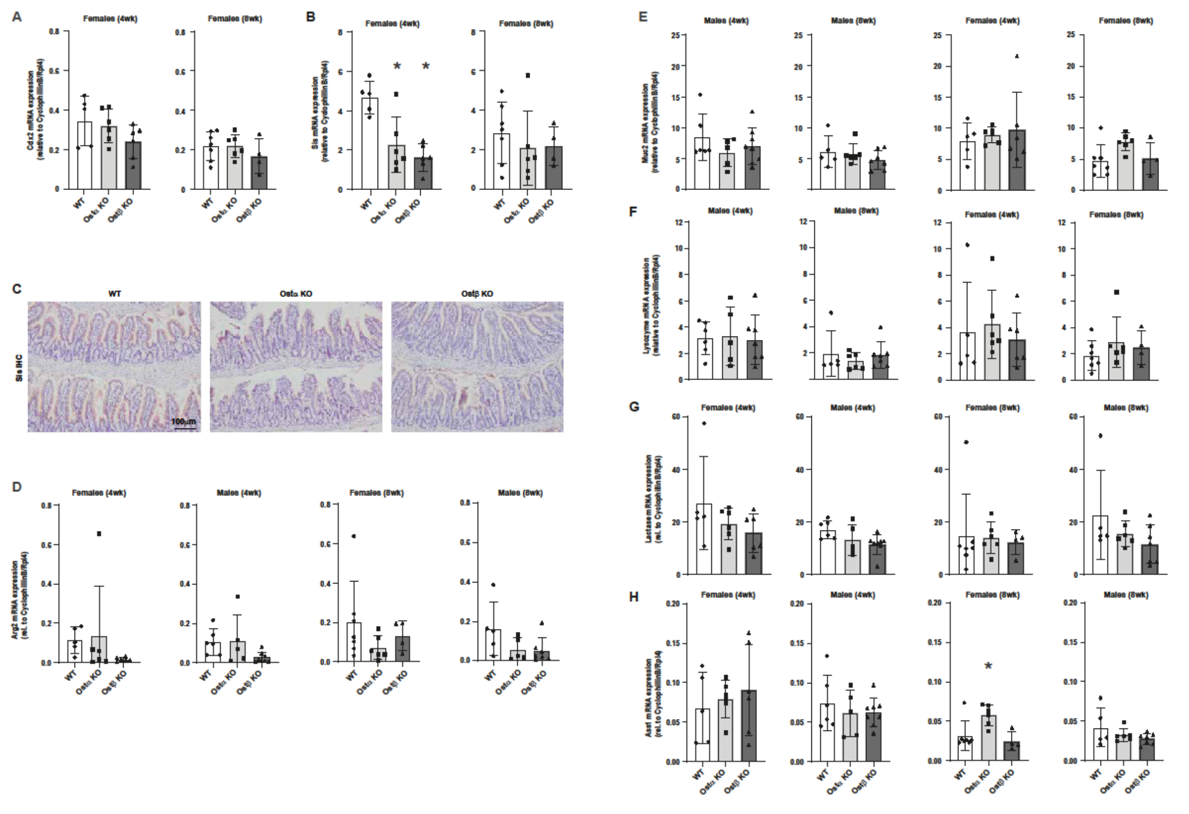

Fig. S3. mRNA expression of 4 and 8 week old *Ostα*$^{-/-}$, *Ostβ*$^{-/-}$ and wild-type females of (A) *Cdx2*, (B) *Sis* (C) Representative microscopic pictures of immunohistochemistry on paraffin-embedded sections of ileums from *Ostα*$^{-/-}$, *Ostβ*$^{-/-}$ and wild-type mice stained with anti-sucrase-isomaltase. Original magnification, 100x. Scale bar 100µm. mRNA expression of 4 week old male and female and 8 week old male and female *Ostα*$^{-/-}$, *Ostβ*$^{-/-}$ and wild-type females of (D) *Arg2* (E) *Muc2* (F) *Lysozyme* (G) *lactase* and (H) *Ass1 (*argininosuccinate synthetase 1). Data are normalized using the geometric mean of CyclophillinB and Rpl4. Data are shown as the mean ±SD with individual points shown in dots. Statistical analysis was done using a one-way ANOVA test, and Dunnett's test to compare with wild-type littermates. *A p-value of <0.05 was considered statistically significant from wild-type mice.

Supplementary figure 4

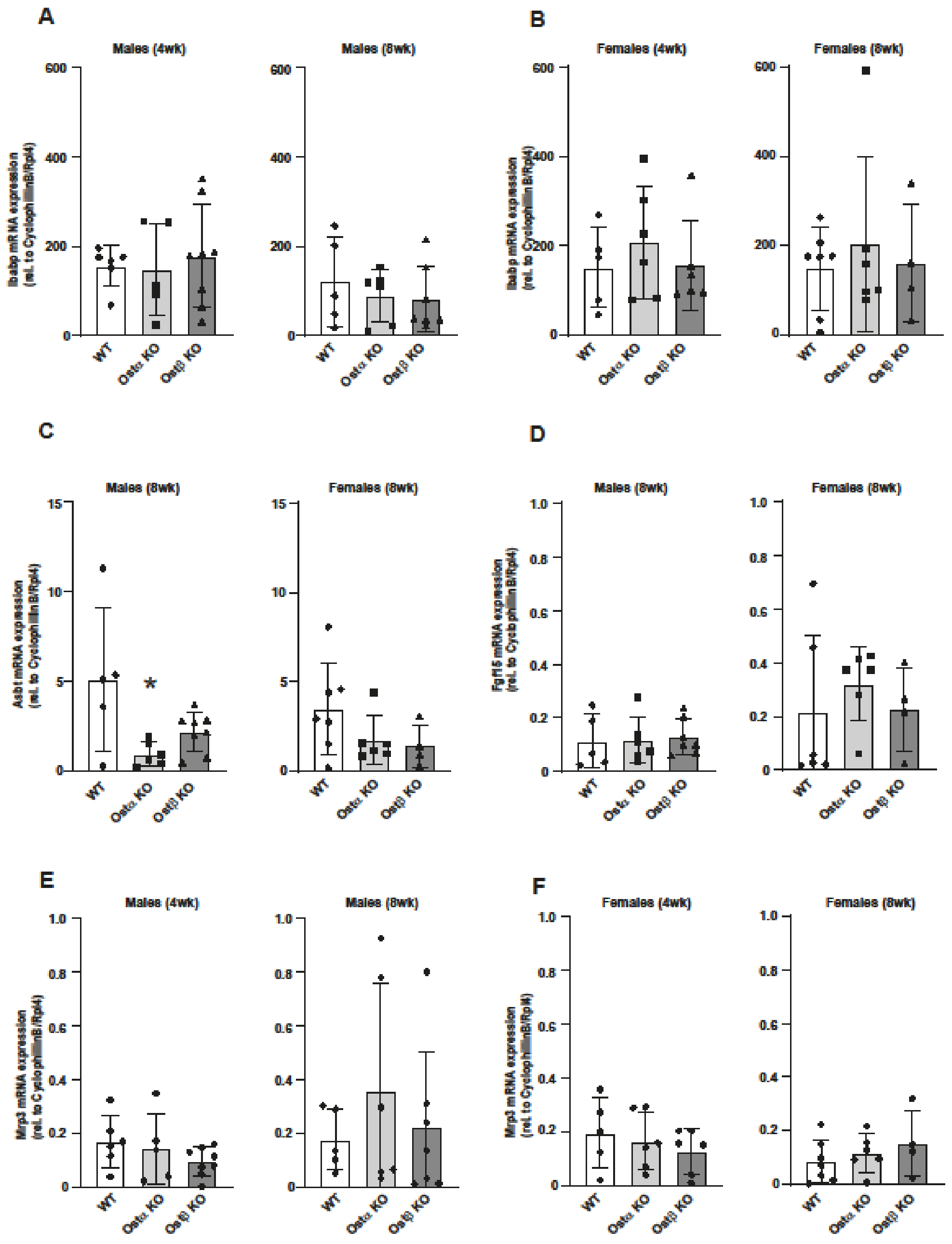

Fig. S4. mRNA expression levels in 4 or 8 week old male and female *Ostα*$^{-/-}$ and *Ostβ*$^{-/-}$ mice. (A) Ibabp expression in males, (B) Fgf15 (C) Asbt expression in males and females (D) *Fgf15* expression in males and females. € Mrp3 expression in mals, and (F) females. Data are normalized using the geometric mean of *CyclophillinB* and *Rpl4*. Data are shown as the mean ±SD with individual points shown in dots (n=4-7). Statistical analysis was done using a one-way ANOVA test, and Dunnett's test to compare with wild-type littermates. *A p-value of <0.05 was considered statistically significant from wild-type mice.

Supplementary Figure 5

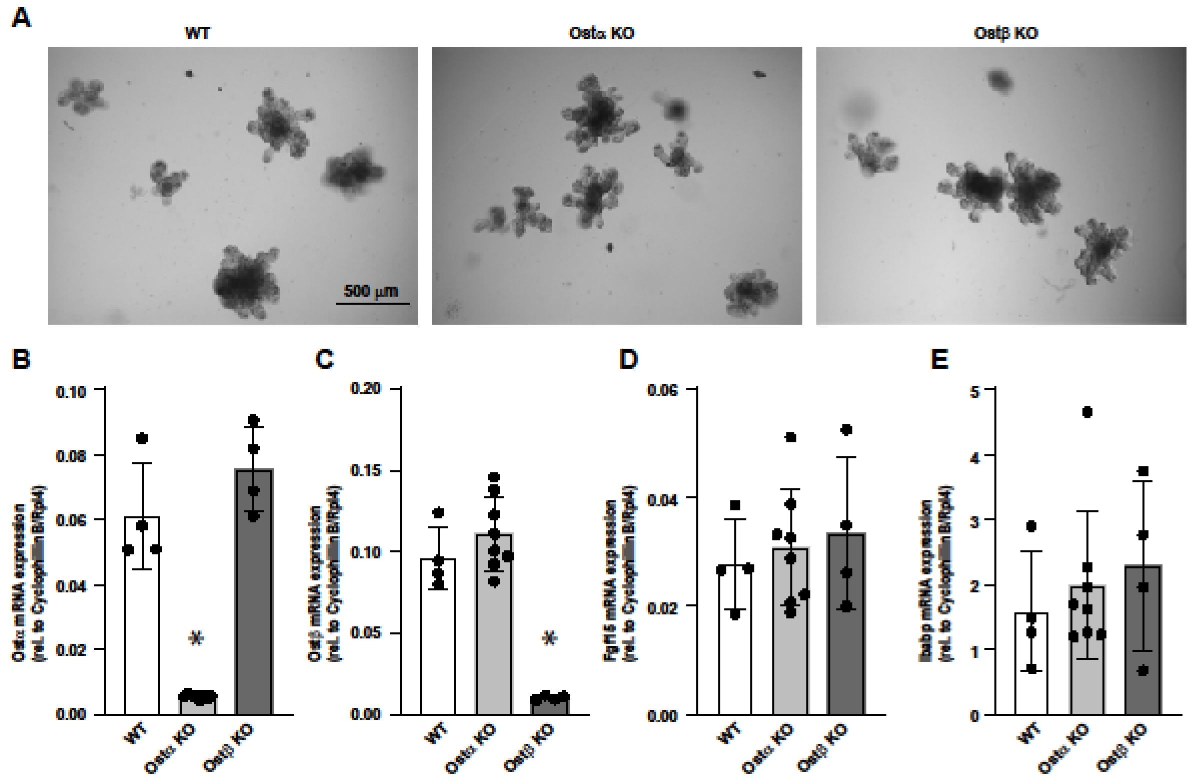

Fig. S5. Ileal Ostα$^{-/-}$, Ostβ$^{-/-}$ and wild-type organoids. (A) Representative microscopic pictures of *Ostα*$^{-/-}$, *Ostβ*$^{-/-}$ and wild-type ileal organoids in culture on day 7 after passaging isolated from eight week old female mice. Original magnification, 50x. Scale bar 500µm. B) mRNA expression of *Ostα* (C) mRNA expression of *Ostβ* (D) mRNA expression of *Fgf15* (E) mRNA expression of *Ibabp*. Data are normalized using the geometric mean of *CyclophillinB* and *Rpl4*. Data are shown as the mean ±SD with individual points shown in dots. Differences between groups were analyzed using a One-way ANOVA test, and a Sidak's multiple comparisons test. *A p-value of <0.05 was considered statistically significant from wild-type mice.

Supplementary Figure 6

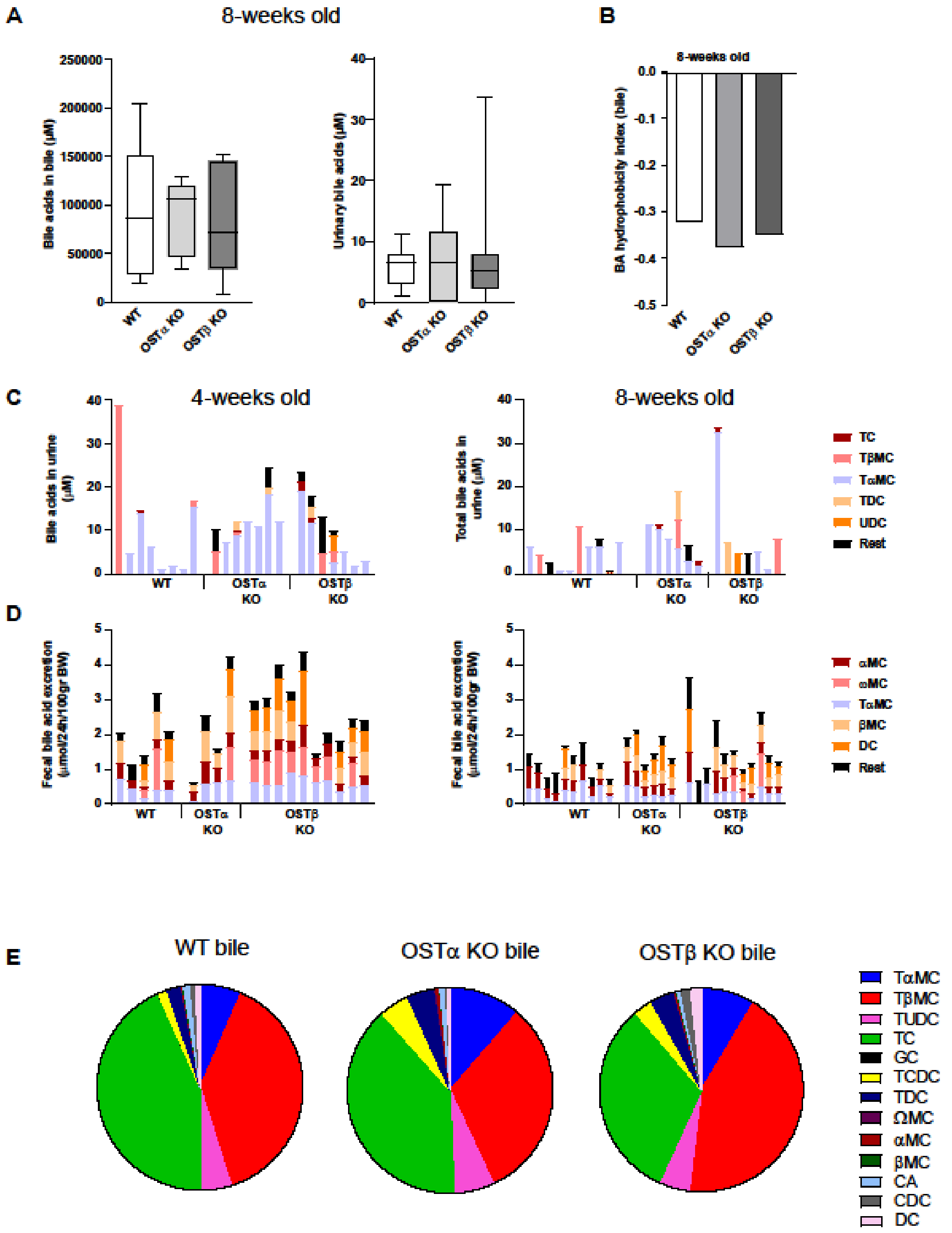

Fig. S6. Concentration and composition of bile acids in circulation and excretory systems of *Ostα*$^{-/-}$, *Ostβ*$^{-/-}$ and wild-type mice, measured by HPLC. (A) Total bile acid concentration in plasma (left) and urine (right) in eight weeks old mice (right) in males and females combined. (B) Hydrophobicity index was similar in bile of *Ostα*$^{-/-}$, *Ostβ*$^{-/-}$ and wild-type mice. (C) Urinary concentration of 17 bile acids is measured, of which the top five is depicted in bile in four weeks old mice (left, n=2-5) and eight weeks old mice (right, n=5-15). (D) Total fecal bile acid excretion per 24 hours per 100gr bodyweight (BW) in four weeks old mice (n=4-10) and eight weeks old mice (n=6-11). (E) Bile acid composition in bile of 8 week old male mice (n=6-11)

Supplementary Figure 7A, B

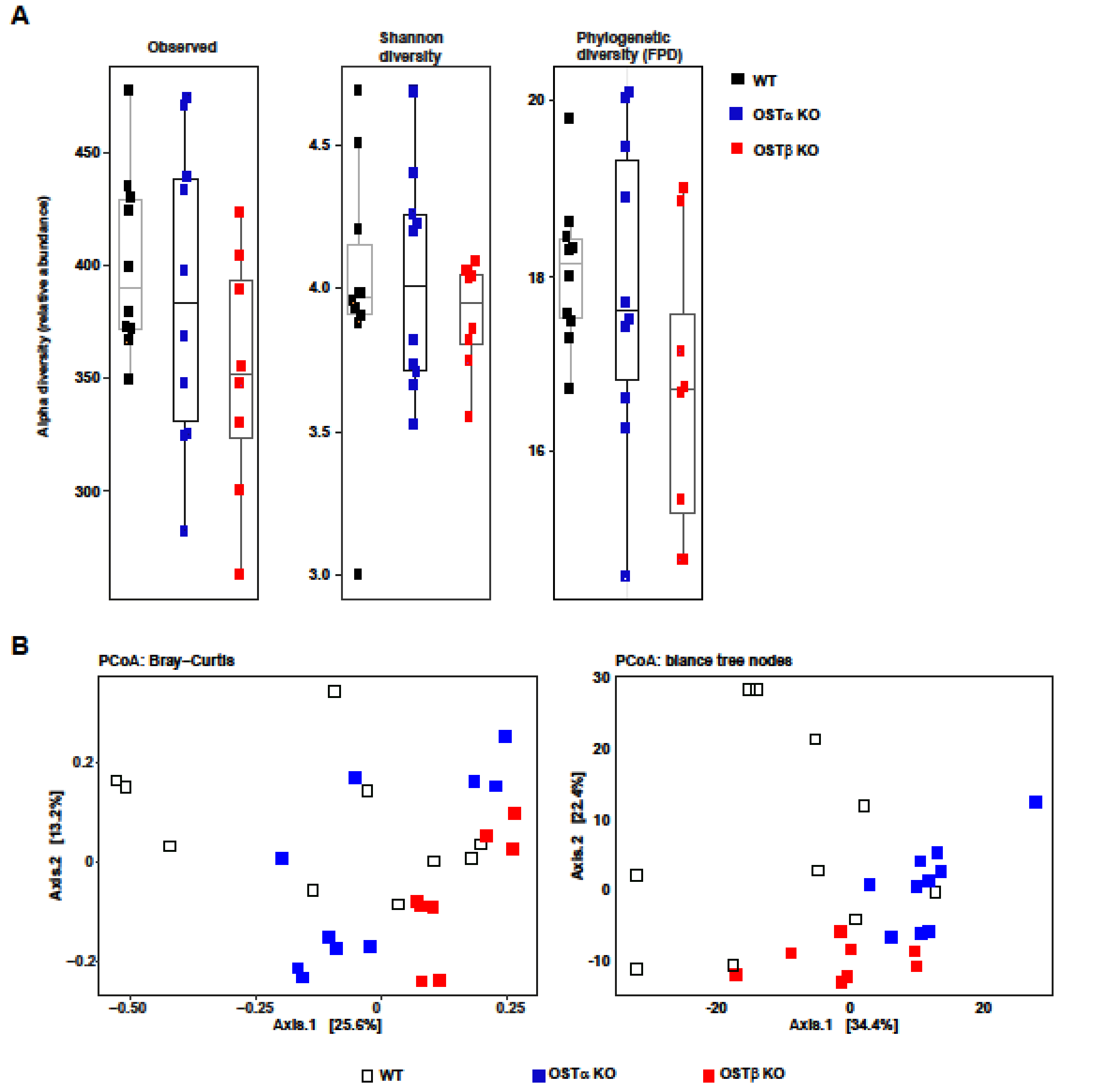

Fig. S7A,B. Ostβ deficiency in mice does not affect α diversity but β diversity decreased in feacal samples. (A) Measures of α diversity. From left to right panel, observed, Shannon and FPD diversity. Not significantly different using ANOVA. The number of observed species is a metric for richness, Shannon diversity indicates evenness and richness, and the phylogenetic diversity (FPD) which indicates genetic diversity. (B) Bray Curtis shown in the Principal Coordinates Analysis ((PERMANOVA p=0.001).

Supplementary Figure 7C

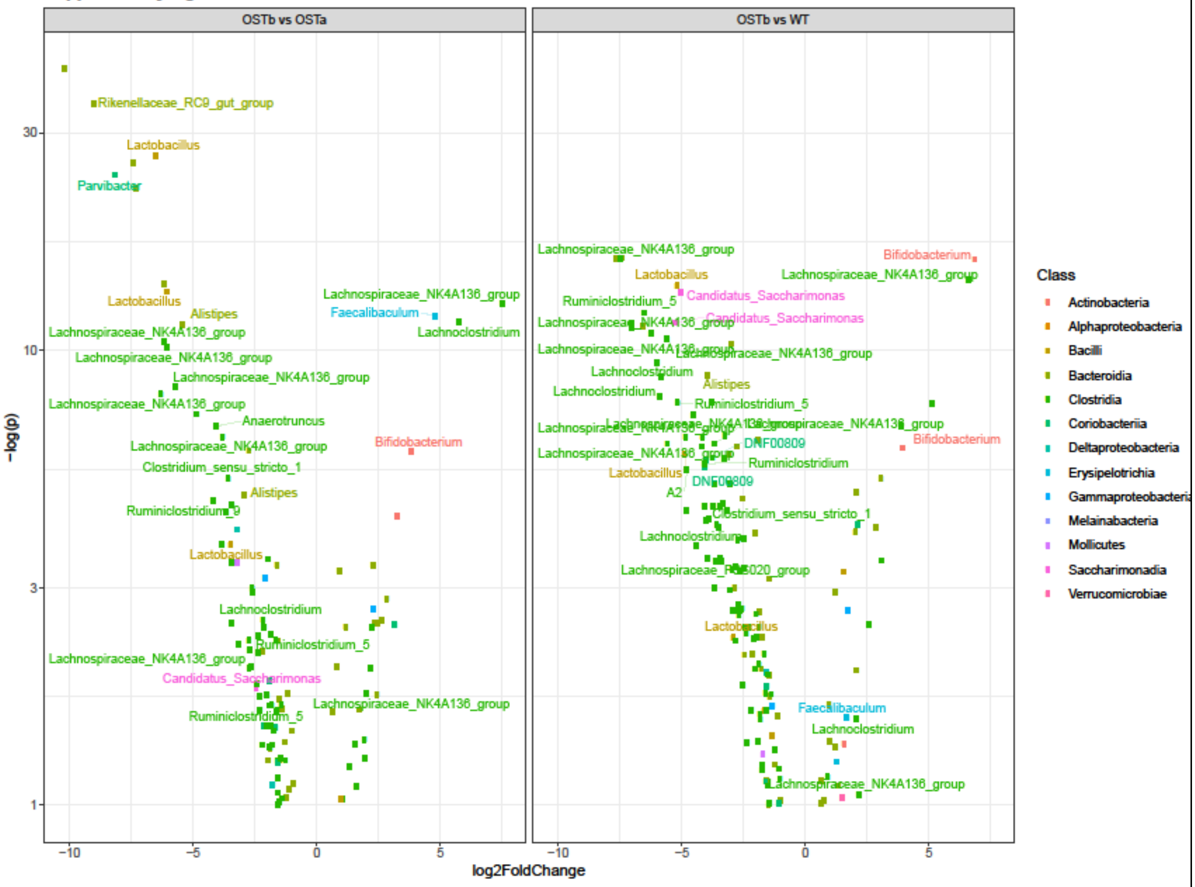

Fig. S7C. Volcano plots of differential abundant taxa between *Ostβ*$^{-/-}$ and wild-type and between *Ostβ*$^{-/-}$ and *Ostα*$^{-/-}$ mice.

Supplementary Figure 8

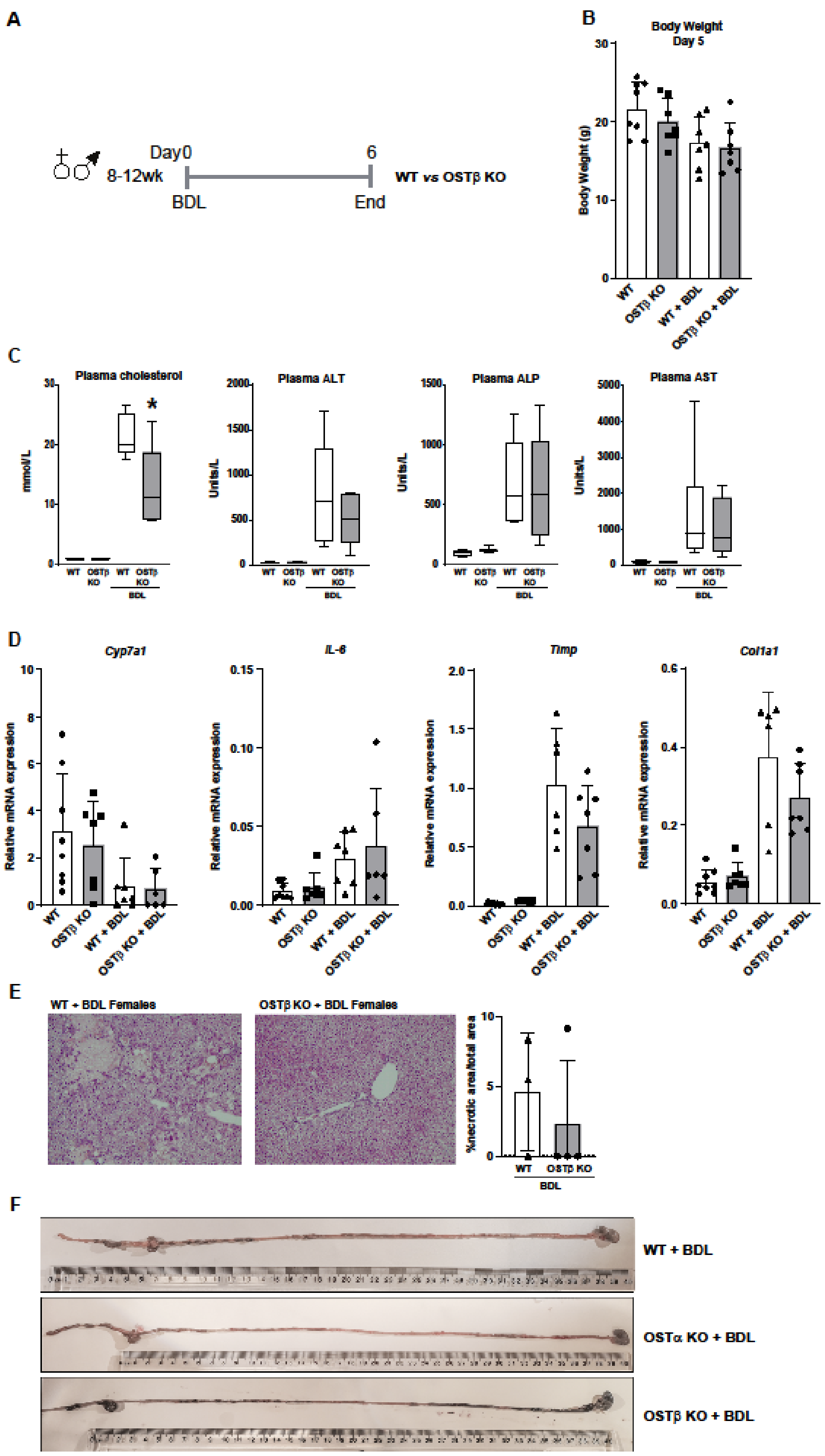

Fig. S8. (A) schematic representation of experimental design where adult *Ostα*$^{-/-}$and *Ostβ*$^{-/-}$ mice were challenged by inducing cholestasis using a common bile duct ligation. (B) Body weight at Day 5 after subjecting male and female adult *Ostβ*$^{-/-}$ mice and wild-type littermates to a common bile duct ligation. (C) Plasma cholesterol, ALT, ALP and AST levels in adult female and male *Ostβ*$^{-/-}$ mice and wild-type littermates 5 days after bile duct ligation. (D) Hepatic mRNA levels of *Cyp7a1, Il-6, Timp* and *Col1a1* in adult female and male *Ostβ*$^{-/-}$

mice and wild-type littermates 5 days after bile duct ligation. (E) Representative liver microscopic pictures of H&E-stained transverse sections (n=3-4 per group) in female mice. Original magnification, 100x. Scale bar 100µm. Areas of necrosis are indicated and quantified (right panel). (F) Representative pictures of the entire intestine in adult male wild-type (n=1), *Ostα*$^{-/-}$(n=3) and *Ostβ*$^{-/-}$ (n=3) mice sacrificed 2 days after bile duct ligation. A student's t-test was used to analyze differences between *Ostβ*$^{-/-}$ mice and wild-type littermates after subjecting them to a common bile duct ligation. *A p-value of <0.05 was considered statistically significant from wild-type mice.

Supplementary Figure 9

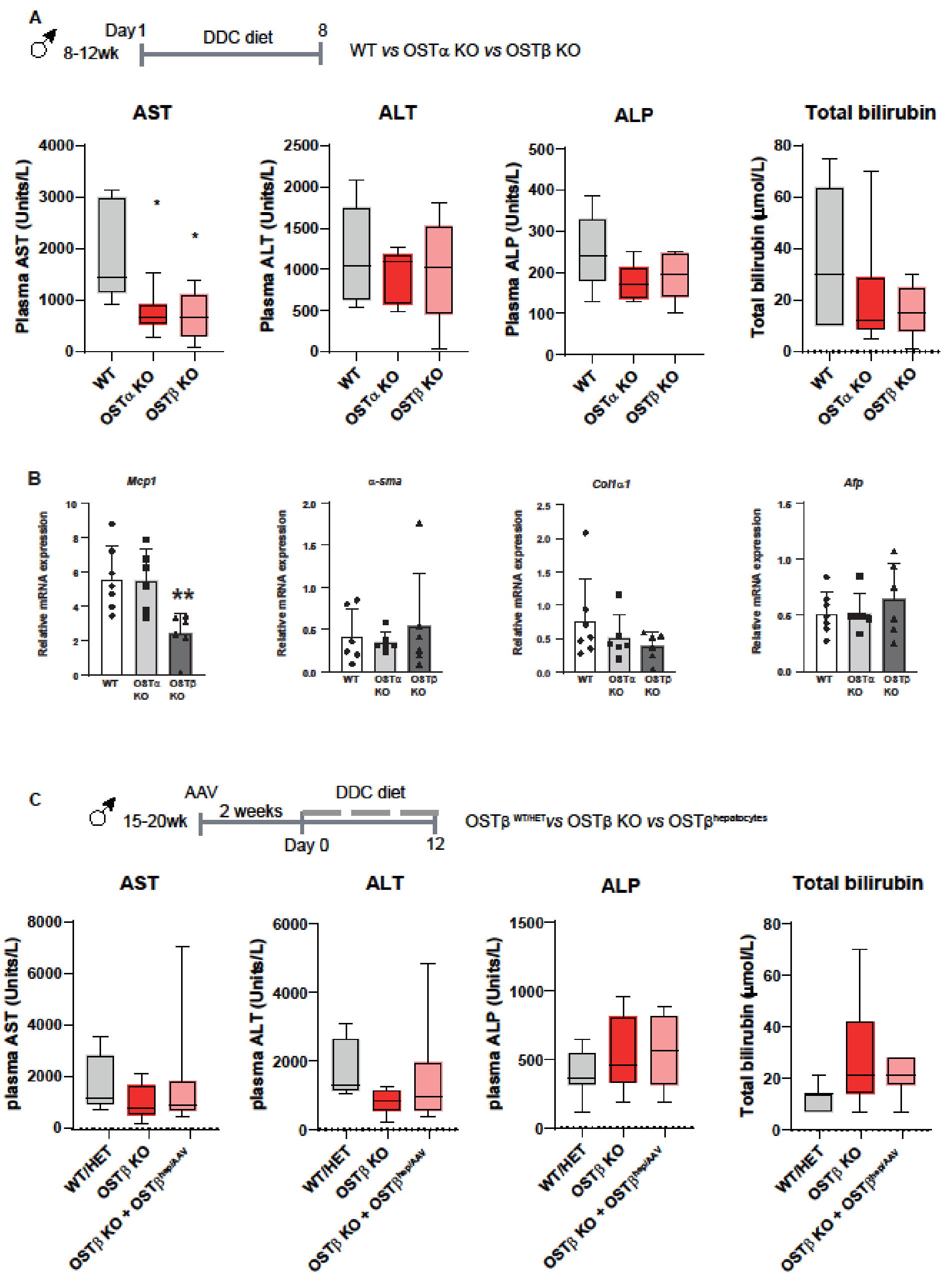

Fig. S9. Challenging adult $Ost\alpha^{-/-}$ and $Ost\beta^{-/-}$ mice and their wild-type littermates supplementing chow diet with 0.1% DDC to induce acute cholestasis. (A) Plasma total bilirubin, ALT, ALP and AST levels. (B) Hepatic mRNA levels of *Mcp1, α-Sma* and *Afp* in mice fed DDC diet. Statistical analysis was done using a one-way ANOVA test, and Dunnett's test to compare with the wild-type littermates. *A p-value of <0.05 was considered statistically significant from wild-type mice and # statistically significant compared with $Ost\alpha^{-/-}$ mice. (C) Schematic of a second DDC experiment. Male mice received AAV8 encoding codon optimized mouse Ostβ. Two weeks later mice were placed on 0.1% DDC diet. Depicted are plasma AST, ALT, ALP and total bilirubin levels at sacrifice 12 days later.

Table S1.

List of mouse primer sequences used for Real-Time qPCR

Gene	Protein	Forward primer (5'-3')	Reverse primer (5'-3')
Slc51a	OSTα	CAGTGGACATAGCCCTCACC	CAGATACCACCGTGGGTGC
Slc51b	OSTβ	GACCACAGTGCAGAGAAAGC	ATTCCAAGGAGCCGCATCT
Slc51b AAV	OSTβ *codon optimized*	TCAGAGCCGAGGATGCCGCC	CGCTAATCAGGGTTTCCCGC
Fabp6	IBABP	GAGACGTGATTGAAAGGGGA	TTACGCGCTCATAGGTCACA
Fgf15	FGF15	ATACGGGCTGATTCGCTACT	GGCTTGGCCTGGATGAAGAT
Slc10a2	ASBT	CCATGGGGTATCTTCGTGGG	GTTCCCGAGTCAACCCACAT
Abcc3	MRP3	CTGGGTCCCCTGCATCTAC	GCCGTCTTGAGCCTGGATAAC
Ppib	CYCLOPHILLINB	TCGGAGCGCAATATGAAGGT	AAAAGGAAGACGACGGAGCC
Rpl4	RPL4	CCTTCTCCTCTCCCCGTCA	GCATAGGGCTGTCTGTTGTTT
Muc2	MUC2	GCCTGTTTGATAGCTGCTATGTGCC	GTTCCGCCAGTCAATGCAGACAC
Lyz	LYSOZYME	CTGTGGGATCAATTGCAGTG	GAATGCCTTGGGGATCTCTC
Ass1	ASS	CATTGGAATGAAGTCCCGAG	GATTTTGCGTACTTCCCGAT
Lct	LACTASE	TATGCAGGCTACGGCACCGG	GCCAAGTTCTGGCATGCGCC
Arg2	ARG2	TAGGGTAATCCCCTCCCTGC	AGCAAGCCAGCTTCTCGAAT
Sis	SUCRASE-ISOMALTASE	AGGTGTCCGCCTGAGCAAGGT	ATGGACGCCAGCAACAGCCA
Cdx2	CDX2	GAAACCTGTGCGAGTGGATG	TCTGTGTACACCACCCGGTA
Hspa5	GRP78	ACTTGGGGACCACCTATTCCT	ATCGCCAATCAGACGCTCC
Hprt	HPRT	CCTAAGATGAGCGCAAGTTGAA	CCACAGGACTAGAACACCTGCTA
Tbp	TBP	ACCGTGAATCTTGGCTGTAAAC	GGAGCAAATCGCTTGGGATTA

Supplementary references

[1] Rao A, Haywood J, Craddock AL, Belinsky MG, Kruh GD, Dawson PA. The organic solute transporter α-β, Ostα-Ostβ, is essential for intestinal bile acid transport and homeostasis. 2008;105:3891-3896.

[2] Donkers JM, Kooijman S, Slijepcevic D, Kunst RF, Roscam Abbing RL, Haazen L, de Waart DR, Levels JH, Schoonjans K, Rensen PC, Oude Elferink RP, van de Graaf SF. NTCP deficiency in mice protects against obesity and hepatosteatosis. JCI Insight 2019;5.

[3] Navis M, Garcia TM, Renes IB, Vermeulen JLM, Meisner S, Wildenberg ME, van den Brink GR, van Elburg RM, Muncan V. Mouse fetal intestinal organoids: new model to study epithelial maturation from suckling to weaning. Embo Reports 2019;20.

[4] Dawson PA, Hubbert M, Haywood J, Craddock AL, Zerangue N, Christian WV, Ballatori N. The heteromeric organic solute transporter alpha-beta, Ostalpha-Ostbeta, is an ileal basolateral bile acid transporter. Journal of Biological Chemistry 2005;280:6960-6968.

[5] Frankenberg T, Rao A, Chen F, Haywood J, Shneider BL, Dawson PA. Regulation of the mouse organic solute transporter α-β, Ostα-Ostβ, by bile acids. American Journal of Physiology-Gastrointestinal and Liver Physiology 2006;290:G912-G922.

[6] Slijepcevic D, Roscam Abbing RLP, Katafuchi T, Blank A, Donkers JM, van Hoppe S, de Waart DR, Tolenaars D, van der Meer JHM, Wildenberg M, Beuers U, Oude Elferink RPJ, Schinkel AH, van de Graaf SFJ. Hepatic uptake of conjugated bile acids is mediated by both sodium taurocholate cotransporting polypeptide and organic anion transporting polypeptides and modulated by intestinal sensing of plasma bile acid levels in mice. Hepatology 2017;66:1631-1643.

[7] Heuman DM. Quantitative Estimation of the Hydrophilic Hydrophobic Balance of Mixed Bile-Salt Solutions. Journal of Lipid Research 1989;30:719-730.

[8] Fickert P, Zollner G, Fuchsbichler A, Stumptner C, Weiglein AH, Lammert F, Marschall HU, Tsybrovskyy O, Zatloukal K, Denk H, Trauner M. Ursodeoxycholic acid aggravates bile infarcts in bile duct-ligated and Mdr2 knockout mice via disruption of cholangioles. Gastroenterology 2002;123:1238-1251.

[9] Slijepcevic D, Roscam Abbing RLP, Fuchs CD, Haazen LCM, Beuers U, Trauner M, Oude Elferink RPJ, van de Graaf SFJ. Na(+) -taurocholate cotransporting polypeptide inhibition has hepatoprotective effects in cholestasis in mice. Hepatology (Baltimore, Md) 2018;68:1057-1069.

[10] Costea PI, Zeller G, Sunagawa S, Pelletier E, Alberti A, Levenez F, Tramontano M, Driessen M, Hercog R, Jung FE, Kultima JR, Hayward MR, Coelho LP, Allen-Vercoe E, Bertrand L, Blaut M, Brown JRM, Carton T, Cools-Portier S, Daigneault M, Derrien M, Druesne A, de Vos WM, Finlay BB, Flint HJ, Guarner F, Hattori M, Heilig H, Luna RA, van Hylckama Vlieg J, Junick J, Klymiuk I, Langella P, Le Chatelier E, Mai V, Manichanh C, Martin JC, Mery C, Morita H, O'Toole PW, Orvain C, Patil KR, Penders J, Persson S, Pons N, Popova M, Salonen A, Saulnier D, Scott KP, Singh B, Slezak K, Veiga P, Versalovic J, Zhao L, Zoetendal EG, Ehrlich SD, Dore J, Bork P. Towards standards for human fecal sample processing in metagenomic studies. Nat Biotechnol 2017;35:1069-1076.

[11] Kozich JJ, Westcott SL, Baxter NT, Highlander SK, Schloss PD. Development of a dual-index sequencing strategy and curation pipeline for analyzing amplicon sequence data on the MiSeq Illumina sequencing platform. Appl Environ Microbiol 2013;79:5112-5120.

[12] Edgar RC. Search and clustering orders of magnitude faster than BLAST. Bioinformatics 2010;26:2460-2461.

[13] Wang Q, Garrity GM, Tiedje JM, Cole JR. Naive Bayesian classifier for rapid assignment of rRNA sequences into the new bacterial taxonomy. Appl Environ Microbiol 2007;73:5261-5267.

[14] Quast C, Pruesse E, Yilmaz P, Gerken J, Schweer T, Yarza P, Peplies J, Glockner FO. The SILVA ribosomal RNA gene database project: improved data processing and web-based tools. Nucleic Acids Res 2013;41:D590-596.

[15] McMurdie PJ, Holmes S. phyloseq: an R package for reproducible interactive analysis and graphics of microbiome census data. PLOS ONE 2013;8:e61217.

[16] Oksanen J, Blanchet FG, Friendly M, Kindt R, Legendre P, McGlinn D, Minchin PR, O'Hara RB, Simpson GL, Solymos P, Stevens MHH, Szoecs E, Wagner H. vegan: Community Ecology Package

Ordination methods, diversity analysis and other functions for community and vegetation ecologists. 2019 [cited; Available from: https://cran.r-project.org/web/packages/vegan/index.html
[17] Kembel SW, Cowan PD, Helmus MR, Cornwell WK, Morlon H, Ackerly DD, Blomberg SP, Webb CO. Picante: R tools for integrating phylogenies and ecology. Bioinformatics 2010;26:1463-1464.
[18] Love MI, Huber W, Anders S. Moderated estimation of fold change and dispersion for RNA-seq data with DESeq2. Genome Biol 2014;15:550.
