# Supplementary material for: Differential and organ-specific functions of organic solute transporter α and β in experimental cholestasis
Source: JHEP Rep. 2022 Mar 5;4(5):100463. doi: 10.1016/j.jhepr.2022.100463 (PMC9019253; doi:10.1016/j.jhepr.2022.100463)
Supplement: Multimedia component 2 [file mmc2.docx]

**JHEP Reports**

**CTAT methods**

Tables for a “Complete, Transparent, Accurate and Timely account” (CTAT) are now mandatory for all revised submissions. The aim is to enhance the reproducibility of methods.

- Only include the parts relevant to your study
- Refer to the CTAT in the main text as ‘Supplementary CTAT Table’
- Do not add subheadings
- Add as many rows as needed to include all information
- Only include one item per row

**If the CTAT form is not relevant to your study, please outline the reasons why:**

|  |
| --- |

- 1. **Antibodies**

| **Name** | **Citation** | **Supplier** | **Cat no.** | **Clone no.** |
| --- | --- | --- | --- | --- |
| rabbit anti-OSTβ | gift from Paul Dawson[[1](#_ENREF_1)] |  |  |  |
| anti-mouse-OSTα | gift from Paul Dawson[[1](#_ENREF_1)] |  |  |  |
| goat anti-sucrase-isomaltase (A-17) |  | Santa Cruz, | sc-27603 |  |
| rabbit anti-phosphohistone H3 |  | ThermoFisher | PA5-17869 |  |
| poly-HRP goat anti-mouse / rabbit |  | VWR | DPVB110HRP |  |
| BrightVision poly-HRP anti-goat |  | VWR | KDPVG110HRP |  |
| anti-NA/K-ATPase |  | gift from Jan Koenderink (Nijmegen, The Netherlands). |  |  |

- 1. **Cell lines**

| **Name** | **Citation** | **Supplier** | **Cat no.** | **Passage no.** | **Authentication test method** |
| --- | --- | --- | --- | --- | --- |
|  |  |  |  |  |  |

- 1. **Organisms**

| **Name** | **Citation** | **Supplier** | **Strain** | **Sex** | **Age** | **Overall n number** |
| --- | --- | --- | --- | --- | --- | --- |
| Ostα-/- | Generated by Rao *et al*[[2](#_ENREF_2)]. | Jackson Laboratory | (B6.129S6-Slc51atm1Pda/J, JAX stock #009082, | Male & female | 4, 8 and 25-30 week old; indicated in manuscript | 42 |
| Ostβ -/- | Generated by van de Wiel *et al*. | Animal Research Institute Amsterdam | C57BL/6J by precise targeted deletion via CRISPR/Cas9 as described in the supplementary methods section. | Male & female | 4, 8 and 25-30 week old; indicated in manuscript | 87 |
| WT littermates | Generated by van de Wiel *et al*. | Animal Research Institute Amsterdam | C57BL/6J | Male & female | 4, 8 and 25-37 week old; indicated in manuscript | 43 |
| one-cell stage wild-type embryos |  |  |  |  |  |  |

- 1. **Sequence based reagents**

| **Name** | **Sequence** | **Supplier** |
| --- | --- | --- |
| Ketamine |  | Alfasan ref:1711347 |
| Xylazine |  | Sedamun, Dechra, 07768 |
| Alcian Blue |  | Sigma, A3157 |
| Nuclear Fast Red |  | Merck, 5189 |
| MEGAshortscript T7 kit |  | Life technologies |
| MEGAclear kit |  | Life technologies |
| Vector NovaRed (HRP) substrate kit |  | Vector Laboratories, SK-4800 |
| Vectamount |  | Vector Laboratories, SK-4800 |
| Tri Reagent |  | Sigma-Aldrich, T9424-200 |
| RNA Mini Kit |  | Bioline, IS510-B064990, B10-52073 |
| RevertAid Reverse Transcriptase |  | Fermentas, EP0442 |
| cOmplete™, Mini, EDTA-free Protease Inhibitor Cocktail |  | Roche Biochem Reagents |
| 3,5-diethoxycarbonyl-1,4-dihydrocollidine |  | Sigma |

- 1. **Biological samples**

| **Description** | **Source** | **Identifier** |
| --- | --- | --- |
| Intestinal mouse organoids[[3](#_ENREF_3)] | Ostα-/- mice  Ostβ -/- mice  Wt mice littermates | ?? |

- 1. **Deposited data**

| **Name of repository** | **Identifier** | **Link** |
| --- | --- | --- |
|  |  |  |

- 1. **Software**

| **Software name** | **Manufacturer** | **Version** |
| --- | --- | --- |
| CRISPR design tool on the ZiFiT website (http://zifit.partners.org/ZiFiT/) |  |  |
| Zhang laboratory website (http://crispr.mit.edu) |  |  |
| ImageJ (1.50i) |  |  |
| GeNORM |  | v3.5 |
| GraphPad Software Inc |  | version 8.0.2 |

- 1. **Other (*e.g*. drugs, proteins, vectors etc.)**

| sgRNA target sequences | 5’-TAGGCTGCTTCTTTCGATTTCT-3’ | 5’-AAACAGAAATCGAAAGAAGCAG-3’ |
| --- | --- | --- |
| pDR274 gRNA cas9-guide plasmid |  |  |
| BSA I restriction enzyme digestion |  |  |
| DraI enzyme |  |  |
| AAV8 particles/kg encoding codon optimized mouse OST beta | VectorBuilder |  |
|  |  |  |
|  |  |  |

- 1. **Please provide the details of the corresponding methods author for the manuscript:**

| Stan van de Graaf  Meibergdreef 69-71  1105 BK Amsterdam  The Netherlands  +31-20-5668832  k.f.vandegraaf@amsterdamumc.nl |
| --- |

**2.0 Please confirm for randomised controlled trials all versions of the clinical protocol are included in the submission. These will be published online as supplementary information.**

|  |
| --- |

**REFERENCES**

1. Dawson, P.A., et al., *The heteromeric organic solute transporter alpha-beta, Ostalpha-Ostbeta, is an ileal basolateral bile acid transporter.* J Biol Chem, 2005. **280**(8): p. 6960-8.

2. Rao, A., et al., *The organic solute transporter alpha-beta, Ostalpha-Ostbeta, is essential for intestinal bile acid transport and homeostasis.* Proc Natl Acad Sci U S A, 2008. **105**(10): p. 3891-6.

3. Navis, M., et al., *Mouse fetal intestinal organoids: new model to study epithelial maturation from suckling to weaning.* Embo Reports, 2019. **20**(2).
